# ATPaseTb2, a Unique Membrane-bound FoF1-ATPase Component, Is Essential in Bloodstream and Dyskinetoplastic Trypanosomes

**DOI:** 10.1371/journal.ppat.1004660

**Published:** 2015-02-25

**Authors:** Karolína Šubrtová, Brian Panicucci, Alena Zíková

**Affiliations:** 1 Institute of Parasitology, Biology Centre, CAS, České Budějovice, Czech Republic; 2 Faculty of Science, University of South Bohemia, České Budějovice, Czech Republic; University of Edinburgh, UNITED KINGDOM

## Abstract

In the infectious stage of *Trypanosoma brucei*, an important parasite of humans and livestock, the mitochondrial (mt) membrane potential (Δψ_m_) is uniquely maintained by the ATP hydrolytic activity and subsequent proton pumping of the essential F_o_F_1_-ATPase. Intriguingly, this multiprotein complex contains several trypanosome-specific subunits of unknown function. Here, we demonstrate that one of the largest novel subunits, ATPaseTb2, is membrane-bound and localizes with monomeric and multimeric assemblies of the FoF1-ATPase. Moreover, RNAi silencing of ATPaseTb2 quickly leads to a significant decrease of the Δψ_m_ that manifests as a decreased growth phenotype, indicating that the F_o_F_1_-ATPase is impaired. To further explore the function of this protein, we employed a trypanosoma strain that lacks mtDNA (dyskinetoplastic, Dk) and thus subunit a, an essential component of the proton pore in the membrane F_o_-moiety. These Dk cells generate the Δψ_m_ by combining the hydrolytic activity of the matrix-facing F_1_-ATPase and the electrogenic exchange of ATP^4^- for ADP^3^- by the ATP/ADP carrier (AAC). Surprisingly, in addition to the expected presence of F1-ATPase, the monomeric and multimeric F_o_F_1_-ATPase complexes were identified. In fact, the immunoprecipitation of a F_1_-ATPase subunit demonstrated that ATPaseTb2 was a component of these complexes. Furthermore, RNAi studies established that the membrane-bound ATPaseTb2 subunit is essential for maintaining normal growth and the Δψ_m_ of Dk cells. Thus, even in the absence of subunit a, a portion of the F_o_F_1_-ATPase is assembled in Dk cells.

## Author Summary

The presence of the F_o_F_1_-ATP synthase in every aerobic organism suggests that evolution has settled on a basic blueprint for the complex rotary motor capable of synthesizing life’s universal energy currency—ATP. However, compared to yeast and mammalian models of the F_o_F_1_-ATP synthase, several recent studies have reported unique structural and functional features of this complex from organisms representing the clades of Chromalveolata, Archaeplastida and Excavata. One of the most striking cases is observed in trypanosomes, important parasites of humans and animals. Notably, the F_o_F_1_-ATP synthase/ATPase switches from synthesizing ATP in the insect vector life stage to hydrolyzing ATP in their mammalian hosts to generate the essential mitochondrial membrane potential (Δψ_m_). Moreover, this indispensable F_o_F_1_-ATPase contains up to 14 trypanosome-specific subunits. Here we characterize one such novel subunit, ATPaseTb2. We demonstrate that this subunit is crucial for the survival of the infectious stage of trypanosomes, part of the fully assembled F_o_F_1_-complex and it is essential for maintaining the Δψ_m_. Given the enzyme’s irreplaceable function and extraordinary composition, we believe that the F_o_F_1_-ATPase is an attractive drug target.

## Introduction

Trypanosomes are unicellular flagellates from the order Kinetoplastida, which is comprised of some of the most devastating human pathogens in the world. For example, in sub-Saharan Africa, infection from *Trypanosoma brucei gambiense* and *T*. *b*. *rhodesiense* causes Human African Trypanosomiasis, which is almost always fatal if left untreated [1]. The latest WHO reports estimate that there are 10,000 new cases annually in endemic regions. Meanwhile, a third subspecies, *T*. *b*. *brucei*, infects livestock and therefore negatively affects the human population through malnutrition and economic hardships [2,3].

*T*. *brucei* parasites have a complex life cycle, alternating between the mammalian host and the insect vector, a tse-tse fly. During this environmental switch, the protist undergoes rapid and dramatic changes in cell morphology and metabolism [4–6]. In particular, the single mitochondrion undergoes extensive remodelling, which reflects the adaptability of the parasite to consume different carbon sources based on their availability [4]. The procyclic (insect) form (PF) of trypanosomes catabolizes amino acids and maintains a well-developed mitochondrion with abundant cristae, Krebs cycle enzymes and a complete oxidative phosphorylation pathway. This pathway includes enzymatic complexes that generate a mitochondrial (mt) membrane potential (Δψ_m_) that is coupled to ATP synthesis by the F_o_F_1_-ATP synthase [5]. In contrast, the bloodstream form (BF) of this parasite populates the glucose-rich fluids (e.g. blood and spinal fluid) of its vertebrate host, allowing them to utilize just glycolysis for ATP production. This results in a drastically reduced mitochondrion that lacks significant cristae, key enzymes of the Krebs cycle and the cytochrome-containing respiratory complexes that pump protons into the inner mt membrane space [6,7]. Despite this reduction, the BF mitochondrion is still an active organelle, holding vital processes e.g. lipid metabolism [8], ion homeostasis [9], calcium signalling [10,11], FeS cluster assembly [12] and acetate production for *de novo* lipid biosynthesis [13]. Importantly, in the absence of proton-pumping respiratory complexes III and IV, the indispensable Δψ_m_ is sustained mainly by the hydrolytic activity of the F_o_F_1_-ATPase. Thus, this complex possesses an essential, unique and irreplaceable function in BF mitochondria [14].

In other eukaryotes, this reverse activity of the F_o_F_1_-ATP synthase is observed only rarely, for very brief moments of time and under very specific conditions (i.e. during oxygen deprivation or in response to damaged or mutated mt respiratory proteins). When the function of the respiratory complexes is compromised, the Δψ_m_ falls below a physiological threshold and is restored by the reverse proton pumping activity of the F_o_F_1_-ATPase, which is powered by ATP hydrolysis. The hydrolytic activity of the catalytic F_1_-ATPase is also essential for exceptional cells that lack mtDNA (ρ° cells). These cells do not express several core subunits of the membrane embedded F_o_-moiety (subunits 6, 8 and 9 in yeast, subunits a and A6L in bovine) of the F_o_F_1_-ATPase, notably those that are components of the proton pore. Thus, the matrix protruding F_1_-ATPase energizes the inner mt membrane by coupling ATP hydrolysis with the exchange of ADP^3-^ for ATP^4-^ by the ATP/ADP carrier (AAC) [15]. The same mechanism for producing the Δψ_m_ is utilized by trypanosomes that lack a mt genome, which is called a kinetoplast [16]. These naturally occuring dyskinetoplastic forms (Dk) of *T*. *brucei* (e.g. *T*. *b*. *evansi* or *T*. *b*.*equiperdum*) cause economically significant diseases in horses, camels, and water buffaloes. Remarkably, these parasites are not able to switch to the insect stage and are transmitted mechanically by bloodsucking insects or by coitus [17]. The mtDNA-lacking trypanosomes can also be chemically induced in the laboratory (e.g. Dk *T*. *brucei* EATRO164) [18]. Interestingly, each of the Dk cell lines characterized so far, bear one of several different compensatory mutations in the nuclear encoded subunit γ that enable the Δψ_m_ to be generated independently of the F_o_-moiety [14,16,19].

In general, the F_o_F_1_-ATP synthase complex consists of two functionally distinct enzymatic segments: the hydrophilic F_1_ catalytic moiety and the membrane-bound F_o_ pore. Both of these subcomplexes are linked together by the central and peripheral stalks. The central stalk rotates with the c-ring when protons are allowed to pass through the F_o_ pore, located between the c-ring and subunit a. In contrast to the rotation of the central stalk, the stationary peripheral stalk plays a crucial role in keeping the catalytic F_1_ headpiece static, thus resisting the rotational torque. The eubacterial F_1_-moiety consists of the catalytic domain and the central stalk, which are comprised of five subunits in a stoichiometry of α_3_, β_3,_ γ_1,_ δ_1,_ ε_1_. The F_o_-moiety is composed of the oligomeric c_10–15_ ring and a single subunit a joined together with two copies of subunit b, which extend from the membrane and form the base of the peripheral stalk. The composition of the eukaryotic enzyme has been determined mainly from detailed studies of F_o_F_1_-ATP synthase purified from the mitochondria of *Bos taurus* and *Saccharomyces cerevisiae*, members of the clade Opisthokonta. These enzymes contain homologous prokaryotic-like core components, but also incorporate additional eukaryotic specific subunits involved in the structure, oligomerization and regulation of the complex [20]. These novel subunits have been assigned to various regions of the F_o_F_1_-ATP synthase: i) subunit ε and inhibitory factor 1 (IF_1_) bind to the F_1_-moiety; ii) the small hydrophobic subunit 8 (subunit A6L in bovine) is located in the membrane interacting with subunit a; iii) the soluble subunit h (subunit F_6_ in bovine), oligomycin sensitivity-conferring protein (OSCP) and hydrophilic subunit d are assigned to the peripheral stalk; iv) subunits e, f, g, all containing one transmembrane helix, are regarded as accessory peripheral stalk proteins that predominantly reside in the inner membrane where they presumably stabilize the utmost hydrophobic subunit 6 (or subunit a in bovine), thereby assisting the peripheral stalk with its stator function [21–23].

Recently, however, several studies have reported unique structural and functional features of the F_o_F_1_-ATP synthase from medically relevant parasites and other organisms that represent the clades of Chromalveolata [24], Archaeplastida [25] and Excavata [26]. In *Plasmodium falciparum*, a member of the phylum Apicomplexa, the genomic data indicates the likely presence of all eukaryotic F_1_ subunits and some of the F_o_ and stator subunits [27]. Conspicuously absent are the subunits a and b, which are crucial to the function of all known F_o_F_1_-ATP synthases. These subunits are also missing in the ATP synthase of *P*. *yoelii* and *Tetrahymena thermophila*, the latter a member of the sister phylum Ciliophora, both of which have been shown experimentally to possess ATP synthase activity [28–30]. This suggest that ciliates and apicomplexan species employ highly divergent or novel subunits to fulfil the functions of the classical subunits a and b. Another example of an unusual F_o_F_1_-ATP synthase complex comes from studies of the chlorophycean algea *Chlamydomonas reinhardtii* and *Polytomella* sp. that determined the complex contains up to 9 unique subunits (Asa1-Asa9) that either form an innovative peripheral stator or are responsible for complex dimerization [31].

Trypanosoma F_o_F_1_-ATP synthase consists of the well conserved F_1_-moiety comprised of subunits α, β, γ_,_ δ_,_ ε and the trypanosome-specific subunit p18 [26,32], and the less characterized F_o_ pore and peripheral stalk where only subunits c, a and OSCP were identified at the gene or protein level [26,33]. Additionally, the complex contains up to 14 Kinetoplastida-specific subunits that lack homology to any of the previously described subunits. Therefore, their position within the complex and their function is unknown. Interestingly, these recently identified F_o_F_1_-ATP synthase subunits specific for *T*. *brucei* (ATPaseTb) may represent early evolutionary attempts to create the functional and structural components of the eukaryotic accessory subunits for this early divergent species. Previously, two of these novel subunits, ATPaseTb1 (Tb10.70.7760) and ATPaseTb2 (Tb927.5.2930), were shown to be essential for the proper function and structural integrity of F_o_F_1_-ATP synthase in the procyclic form of this parasite [26].

Here, we have extended this study and functionally characterized ATPaseTb2 in the disease-relevant stage of *T*. *brucei* and Dk *T*. *b evansi*. Our results demonstrate that the membrane-bound ATPaseTb2 protein is indispensable for the survival of BF and Dk trypanosomes because it is crucial for maintaining the Δψ_m_ in these cells. Furthermore, the unexpected observation of assembled F_o_F_1_-ATPase complexes in two different strains of trypanosomes lacking mt DNA suggests that the composition of the Dk F_o_F_1_-ATPase complex is more similar to the BF F_o_F_1_-ATPase complex than previously thought.

## Results

### Bioinformatics reveals that ATPaseTb2 contains a putative transmembrane domain and a region homologous to the bovine subunit d

**Fig 1:**
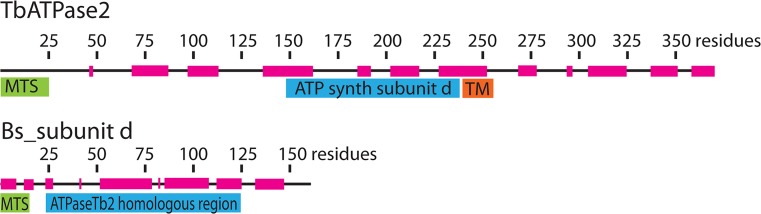
Bioinformatics reveal that ATPaseTb2 possesses a region of low homology to ATPase subunit d. A schematic representation depicting the ATPaseTb2 protein (370 aminoacids (AA)) and the *Bos taurus* subunit d (Bs_sub d, 161 AA) was created using Adobe Illustrator CS5.1. The mitochondrial targeting signals (MTS, green) were predicted by Mitoprot II v1.101. The transmembrane domain (TM, orange) within the ATPaseTb2 sequence was predicted by MEMSAT-SVM software. The homologous region (blue) to Bs_sub d was determined using HHpred toolkit. The regions of alpha-helices (magenta) depicted on the amino acid sequence line were predicted by software from EMBOSS garnier.

ATPaseTb2 is annotated as a hypothetical protein in the TriTrypDB database (www.tritrypdb.org) and its mt localization was predicted by the presence of an N-terminal mt targeting sequence assigned by Mitoprot II v1.101 [34] (probability 0.823). Strikingly, this subunit has no detectable homologs outside Kinetoplastida based on a similarity sequence search (e.g. BLAST). To uncover any homologous relationships for protein sequences that do not share high enough sequence identity, but might have similar secondary structure, we employed the HHpred toolkit (http://toolkit.tuebingen.mpg.de), which is based on an HMM-HMM comparison that reveals homologous relationships even if the sequences share less than 20% sequence identity [35]. All of the available Kinetoplastida ATPaseTb2 homologs obtained from the TriTrypDB (S1A Fig.) were analyzed using HHpred software. Among the first five structural homologs detected was the bovine F_o_F_1_-ATP synthase subunit d, with a probability of 44.7 and a P-value of 0.0021. Then a consensus sequence generated from an alignment of this 92 amino acids (aa) region from all identified kinetoplastida homologs was resubmitted for HHpred analysis. This search returned a significant hit to the bovine subunit d with a probability of 78.1 and a P-value of 0.0002 (S1B Fig.). This bovine F_o_F_1_-ATP synthase subunit is comprised of 160 aa and resides within the peripheral stalk where it interacts with all the other three components of the peripheral stalk (b, F_6_ and OSCP). This subunit is predominantly hydrophilic, contains several long alpha-helices and is essential for the enzymatic function [23,36,37]. The region of similarity to subunit d falls in the middle of the ATPaseTb2 sequence and it is followed by a putative trans-membrane (TM) domain (aa 239–254) that was predicted by MEMSAT-SVM software (http://bioinf.cs.ucl.ac.uk/psipred/) [38] with a -0.738 prediction score (Fig. 1). This prediction is intriguing because a typical eukaryotic subunit d is not directly attached to the membrane, but rather it interacts with the soluble region of the membrane-bound subunit b via three coiled-coil interactions [23]. Thus, ATPaseTb2 may represent a novel subunit of the membrane-bound peripheral stalk that possibly depicts an early divergent functional alternative of the stator by creating a chimera of subunits b and d.

### ATPaseTb2 is a mt membrane-bound protein expressed in various forms of *T*. *brucei*

**Fig 2:**
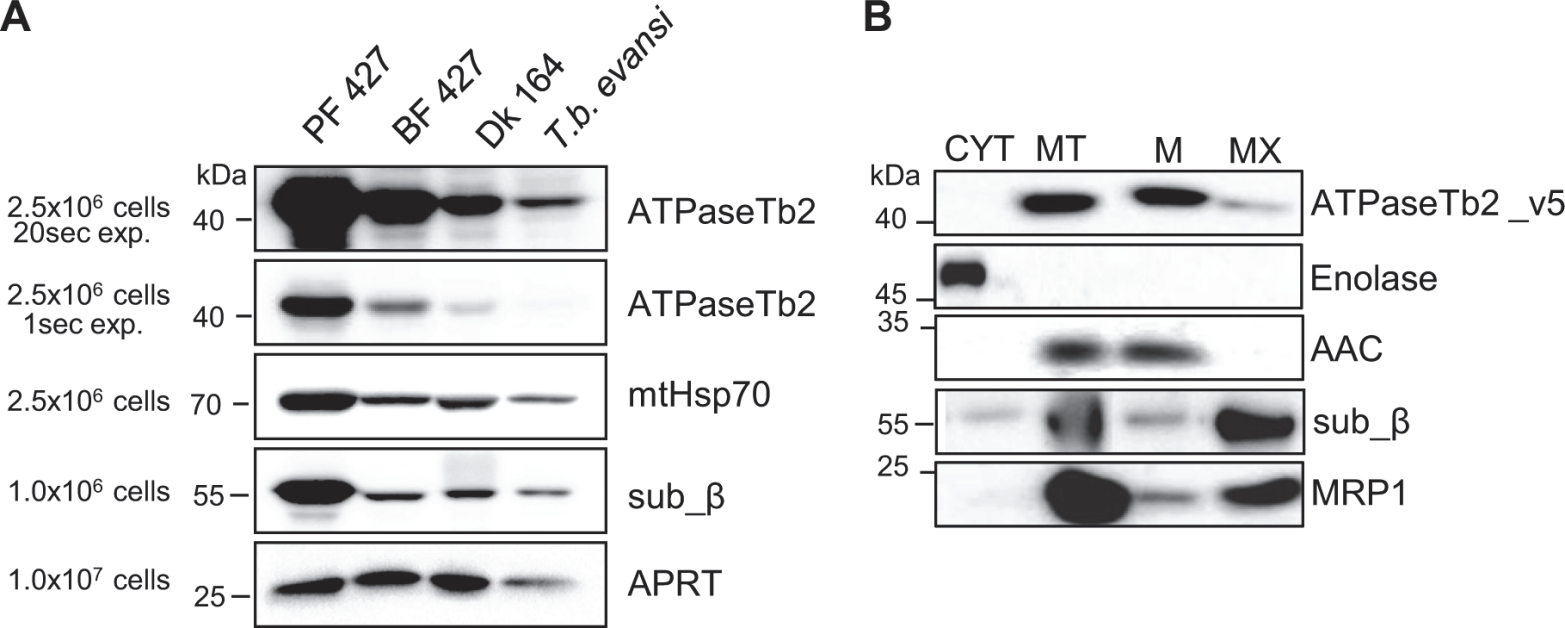
ATPaseTb2 is expressed in PF427, BF427, Dk164 *T*. *brucei* and *T*. *b*. *evansi* cells and it is a membrane-bound protein. A) The steady state abundance of ATPaseTb2 (two different exposure times), subunit β, mtHsp70 and cytosolic adenosine phosphoribosyl transferase (APRT) was determined in PF427, BF427, Dk164 and *T*. *b*. *evansi* cells by western blot analysis of whole cell lysates from 2.5x10^6^ cells or 1x10^6^ cells (for subunit β and APRT) resolved on SDS-PAGE gels. The pertinent sizes of the protein marker are indicated on the left. B) Subcellular localization of ATPaseTb2 was determined using BF cells expressing v5-tagged ATPaseTb2. Cytosolic (CYT) and mitochondrial (MT) fractions were obtained by hypotonic lysis. Mt pellets were further treated with Na_2_CO_3_ and spun at a high-speed to obtain mt membrane (M) and matrix (MX) fractions. Purified fractions were analyzed by western blot with the following antibodies: anti-v5 (ATPaseTb2_v5), anti-enolase (cytosol), anti-AAC (mt inner membrane), anti-β (F_1_-ATPase subunit) and anti-MRP1 (mt matrix). The relevant sizes of the protein marker are indicated on the left.

The mitochondrial localization, as well as the structural and functional association of ATPaseTb2 with the F_o_F_1_-ATP synthase in PF *T*. *brucei* cells was previously described [26]. Because the activity of this complex significantly differs between the insect and mammalian stage of the parasite, we were interested if the ATPaseTb2 protein is also expressed in trypanosomes residing in the bloodstream of their host. Therefore, we cultured PF427 (insect stage), BF427 (bloodstream forms) and the laboratory-induced dyskinetoplastic Dk164 *T*. *brucei* cells, in addition to the naturally occurring Dk *T*.*b*.*evansi*. Whole cell lysates from PF427, BF427, Dk164 and *T*.*b*.*evansi* cells were fractionated by SDS-PAGE and analyzed by western blot using a specific polyclonal antibody against ATPaseTb2 (Fig. 2A). Interestingly, ATPaseTb2 is expressed in all four cell types; however, its expression is significantly decreased in BF427 and even more reduced in Dk164 and *T*.*b*.*evansi* cells. A similar expression pattern for F_1_-ATPase subunit β and mt chaperone Hsp70 was also observed between PF, BF and Dk cells (Fig. 2A). These results are in agreement with the proposed reduction of the mitochondria size, function and activity in the various bloodstream forms of *T*. *brucei* [39].

To experimentally investigate the subcellular localization of ATPaseTb2 within the bloodstream *T*. *brucei* cell, the ATPaseTb2 gene product was C-terminally tagged with a v5-tag and inducibly expressed using tetracycline (tet). Cytosolic and mitochondrial fractions were isolated from a hypotonic lysate of cells over-expressing the ATPaseTb2_v5 protein. The mitochondrial fractions were then treated with digitonin to create mitoplasts that were further incubated with sodium carbonate (Na_2_CO_3_) to obtain mt membrane and mt matrix fractions. The subsequent western analyses established the purity of the extracted fractions as the cytosolic enolase, the mt inner membrane carrier protein (AAC) and the matrix-localized guide RNA binding protein (MRP1) were confined within their respective subcellular fractions. Notably, ATPaseTb2 was predominantly localized in the mt membrane fraction, while subunit β of the matrix-soluble F_1_-ATPase was released into the matrix fraction (Fig. 2B), demonstrating that ATPaseTb2 is firmly embedded in the inner mt membrane.

### ATPaseTb2 is a bona fide subunit of F_o_F_1_-ATPase monomers and higher oligomers

**Fig 3:**
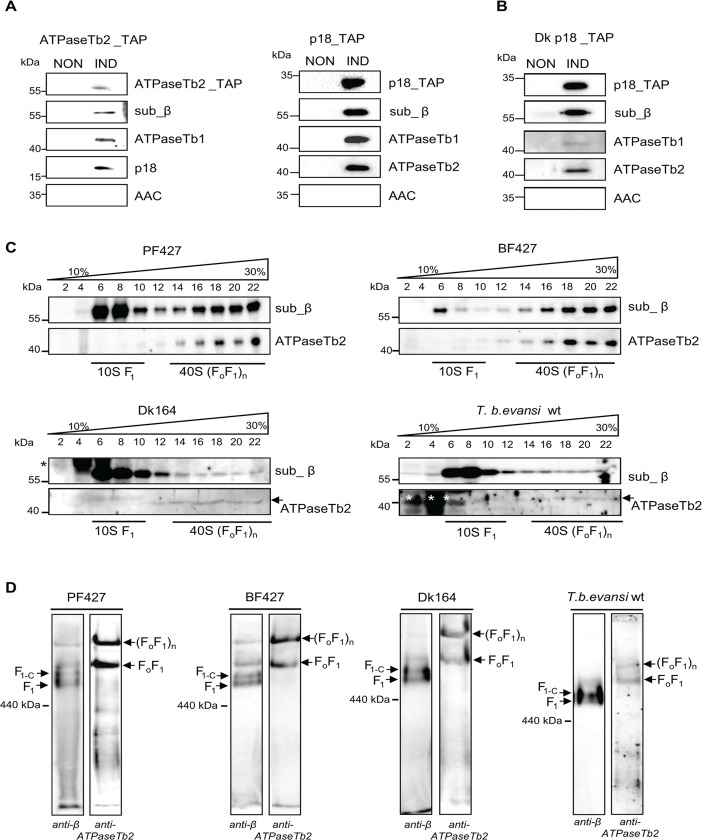
ATPaseTb2 is a bona fide subunit of the monomeric and multimeric F_o_F_1_-ATPases. A) ATPaseTb2_TAP and p18_TAP tagged complexes were purified from BF cells using one-step IgG affinity chromatography from non-induced (NON) and 2 days induced (IND) cells containing the regulatable ectopic TAP tagged protein. The tagged protein complexes were eluated by TEV protease, fractionated on SDS-PAGE and examined by western blot analyzes. The presence of the tagged ATPaseTb2 and p18 subunits was verified using a *c*-myc antibody (top panels: ATPaseTb2_TAP and p18_TAP). The known F_o_F_1_-ATPase subunits (sub β, p18 and ATPase_Tb1) were detected using specific antibodies. The lack of signal for AAC serves as a control for specificity of the used method. The applicable sizes of the protein marker are indicated on the left. B) p18_TAP tagged complexes were purified from dyskinetoplastic *T*. *b*. *evansi* as described above for BF cells and subjected to the same set of antibodies. C) The sedimentation profile of F_1_- and F_o_F_1_-ATPase complexes was determined using glycerol gradient sedimentation. Hypotonically purified mitochondria from PF427, BF427, Dk164 and *T*. *b*. *evansi* cells were lysed with 1% Triton X-100 and fractionated on a 10–30% glycerol gradient. The glycerol gradient fractions were collected, fractionated by SDS-PAGE and analyzed by western blots. Western analyzes with an anti-β antibody depicted the sedimentation profile of the F_1_-ATPase and monomeric/multimeric F_o_F_1_-ATP synthase complexes, whereas the anti- ATPaseTb2 antibody only immunodecorates this protein within the monomeric/multimeric F_o_F_1_-ATPase complexes. The subdivision of the various structural forms of the F_o_F_1_-ATPase complexes are underlined as determined in [26]. The glycerol gradient fractions and the sizes of the protein marker are indicated. Nonspecific bands visible in Dk164 and *T*. *b*. *evansi* gradients are indicated by asterisks. D) The native F_1_- and F_o_F_1_-ATPase complexes were visualized using hrCNE. Purified mitochondria from PF427, BF427, Dk164 and *T*.*b*.*evansi* cells were lysed with digitonin (4 mg/mg), fractionated on a 3%-12% hrCNE and blotted onto a nitrocellulose membrane. The F_1_-ATPase (F_1_), the F_1_-ATPase bound with the c-ring (F_1+C_) and the monomeric F_o_F_1_/multimeric (F_o_F_1_)_n_ complexes were all visualized using specific polyclonal antibodies against either subunit β or ATPaseTb2. The size of ferritin from the equine spleen (440 kDa) is indicated on the left.

To confirm that ATPaseTb2 is a genuine subunit of both the BF and Dk F_o_F_1_-ATPase, we employed the same strategy applied previously for PF cells [26]. Therefore, we first created BF cell lines in which either the ATPaseTb2 or the F_1_ subunit p18 were inducibly expressed with a C-terminal tag. Complexes assembled with a tagged protein were then purified using a one-step IgG affinity purification, followed by treatment with TEV protease to release bound complexes from the IgG beads. The TEV eluates were further analyzed by western blot for the presence of the tagged proteins, known F_o_F_1_-ATPase subunits, and AAC (Fig. 3A). Importantly, the presence of the tagged subunits ATPase Tb2 and p18 in the respective cell lines were confirmed using a specific antibody against the *c*-myc epitope that comprises part of the tag. In addition, the ATPaseTb2_TAP eluate contained F_o_F_1_-ATPase subunits β, p18 and ATPaseTb1. Vice versa, the p18_TAP eluate contained subunits ATPaseTb2, ATPaseTb1 and subunit β. While AAC has been described to associate with mammalian F_o_F_1_-ATPase under certain conditions [40], it is not a core component of the enzyme and the lack of its signal indicates the stringency of this technique. The TAP tag purification from non-induced cells was used as a negative control to verify that the detected proteins do not bind non-specifically to the charged beads. To analyze the composition of the Dk F_o_F_1_-ATPase, p18 tagged complexes were purified and subjected to the same set of antibodies described for the BF complexes (Fig. 3B). These results also depicted the same interactions seen in Fig. 3A. Therefore, the co-purification of ATPaseTb2 with known ATPase subunits p18 and β validates that it is an authentic constituent of the F_o_F_1_-ATPase complex in both BF and Dk cells.

The sedimentation profile of F_o_F_1_-ATP synthase in 10–30% glycerol gradients (GG) was previously published and revealed two distinct regions representing the F_1_-moiety and the F_o_F_1_-monomer and multimeric complexes [26]. In order to specify if ATPaseTb2 is a component of the F_1_-moiety or the F_o_F_1_-complex, the glycerol gradient sedimentation profile was determined using mt lysates from all four cell types: PF427, BF427, Dk164 and *T*.*b*.*evansi*. In correlation with the literature, a new specific antibody raised against the F_1_-subunit β depicted both regions, which are defined as ∼10S (fractions 6–10), and ∼40S (fractions 14–22) (Fig. 3C). A different sedimentation pattern was revealed when a specific antibody recognizing ATPaseTb2 detected only the 40S region (fractions 14–22), representing the F_o_F_1_-monomer and -multimers. These findings suggest that the novel subunit is not a component of the F_1_-moiety, but rather a member of the fully assembled complex. Furthermore, compelling results hinting at the function of this hypothetical protein were also obtained when the mt lysates of Dk164 and *T*. *b*. *evansi* cells were fractionated on similar gradients. While the majority of the signal for subunit β was predictably detected in fractions 6–10, representing the F_1_-ATPase complex, there was also a weak signal detected in higher S values. These same ∼40S complexes were identified using the ATPaseTb2 antibody (Fig. 3C). This indicates that in the absence of subunit a, the F_1_-moiety is still attached to the mt membrane via a central stalk, a ring of subunit c and the peripheral stalk. Since the F_o_ subunit a is missing in these cells, the attachment of this complex to the membrane is presumably not as strong as in BF cells. Therefore, upon treatment with detergent to lyse the mitochondria, a majority of F_1_-ATPase is released and detected in lower S values while a small portion of the F_o_F_1_-complexes is preserved and sediments at higher S value fractions. Consequently, our data suggests that previously undetected membrane-bound F_o_F_1_-complexes are present in the mitochondria of the Dk trypanosomes.

To verify our intriguing results, the F_o_F_1_-ATP synthase/ATPase complex was examined by an alternative method involving high resolution clear native electrophoresis (hrCNE) followed by western blot analysis. Results obtained using the antibody against subunit β revealed four predominant bands in both the PF427 and BF427 samples, representing various forms of the F_1_-moiety and F_o_F_1_-complexes (Fig. 3D). The lowest band likely corresponds to the F_1_-ATPase (subunits α, β, γ,δ,ε), while the one above it seemingly represents F_1_-ATPase with a ring of subunit c, as it has been described for the mammalian complex [41]. Noticeably, in each sample the antibody against ATPaseTb2 immunodetected only two major bands corresponding to the F_o_F_1_-monomer and -multimers. When the mt lysates of Dk164 and *T*. *b*. *evansi* cells were fractionated on a hrCNE, subunit β was mainly detected in the lower bands representing the F_1_-moiety and the F_o_F_1_-monomer, while only a weaker band was observed at the size depicting F_o_F_1_-multimers. But western blot analysis with the ATPaseTb2 antibody clearly demonstrated the existence of higher assemblies of the F_o_F_1_-ATPase complexes (Fig. 3D). Thus, ATPaseTb2 is a membrane-bound subunit of the monomeric and multimeric F_o_F_1_-ATP synthase/ATPase complex in all four *T*. *brucei* cell types examined. Furthermore, no significant differences in size were observed between the F_o_F_1_-monomeric and -multimeric complexes detected in PF427, BF427 and Dk cells, indicating that subunit a might be the only missing component of the assembled F_o_F_1_-ATPase complex in dyskinetoplastic cells.

### Silencing of ATPaseTb2 inhibits cell growth by decreasing the Δψ_m_ in BF *T*. *brucei*

**Fig 4:**
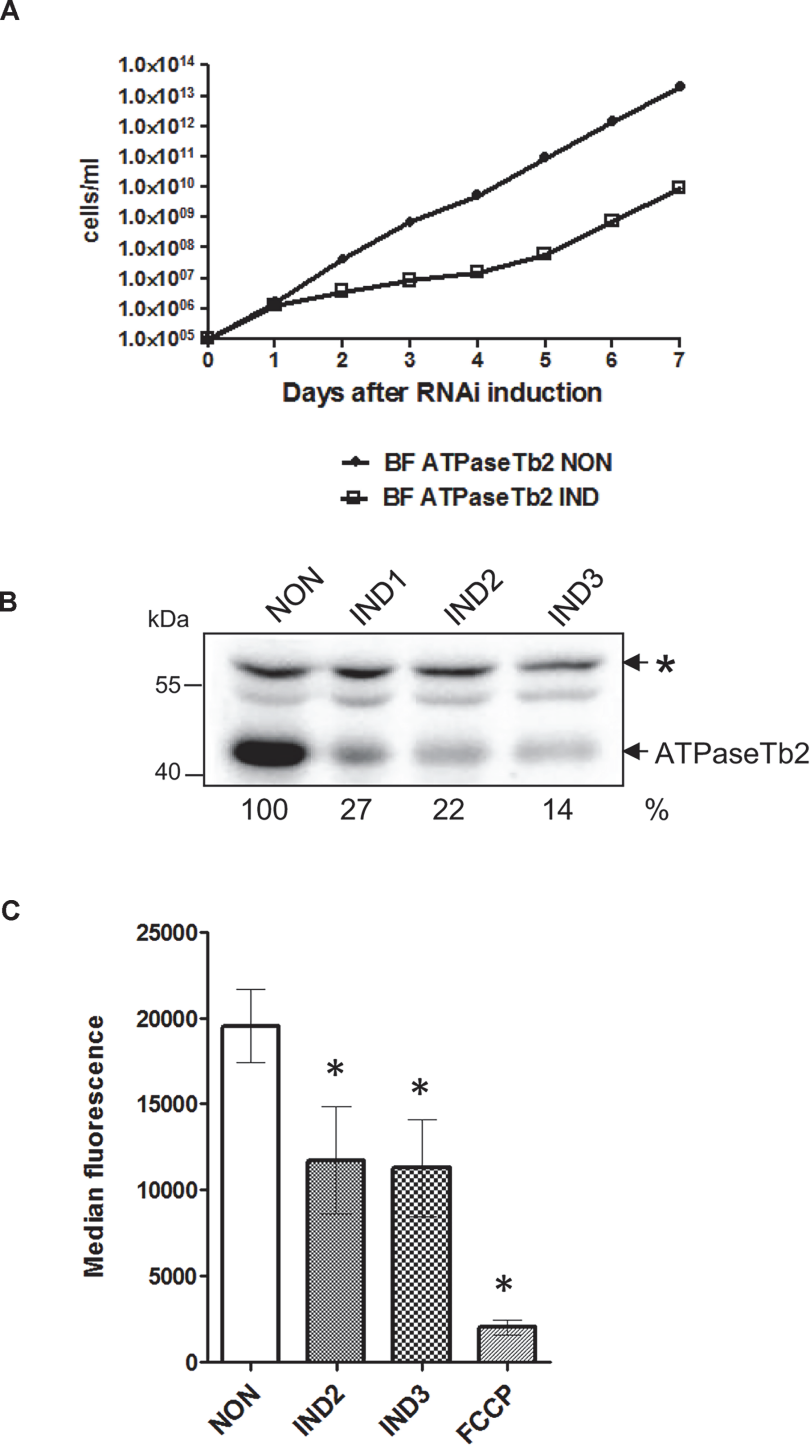
RNAi silencing of ATPaseTb2 inhibits the growth and mt membrane potential of BF *T*. *brucei*. A) Growth curves of the noninduced (NON) and RNAi induced (IND) ATPaseTb2 RNAi bloodstream *T*. *brucei* cell lines were measured for 7 days. Cells were maintained in the exponential growth phase (between 10^5^ and 10^6^ cells/ml) and the cumulative cell number was calculated from cell densities adjusted by the dilution factor needed to seed the cultures at 10^5^ cells/ml each day. The figure is representative of three independent RNAi-inductions. B) The steady-state abundance of ATPaseTb2 in non-induced (NON) RNAi cells and in cells induced with tet for 1, 2 and 3 days (IND1, IND2, IND3) was determined by western blot analysis using a specific ATPaseTb2 antibody. The non-specific band (marked with an asterisk) detected on the same membrane served as a loading control. The numbers beneath the blots represent the abundance of immunodetected ATPaseTb2 expressed as a percentage of the non-induced samples after normalizing to the loading control. The relevant sizes of the protein marker are indicated on the left. The figure is a representative western blot from three independent RNAi-inductions. C) Using Mitotracker Red CMX-Ros, the mt membrane potential was measured by flow cytometry in non-induced (NON) ATPaseTb2 RNAi cells and cells induced for 2 or 3 days (IND2 and IND3). The median fluorescence for each sample is depicted on the y-axis of the column graph. The results are means ± s.d. (n = 3). *P< 0.05, Student’s *t*-test.

To assess the importance of ATPaseTb2 in the infective stage of *T*. *brucei* and to evaluate its functional association with F_o_F_1_-ATPase, an ATPaseTb2 RNA interference (RNAi) cell line was created. The expression of dsRNA was triggered by the addition of tet to the culture medium and the *in vitro* growth of the ATPaseTb2 knock-down (KD) cells was inspected for seven days (Fig. 4A). Strikingly, a significant growth phenotype was already detected in RNAi induced cells on just the second day of tet addition. Presumably, the powerful selection forces acting on these cells missing a critical protein resulted in the emergence of a subpopulation that was no longer responsive to RNAi induction after only 120 hours, leading to the growth recovery of the culture (Fig. 4A). This phenomenon is often reported for RNAi experiments in BF *T*. *brucei* [42].

The targeted KD of ATPaseTb2 was confirmed by western blot analysis of whole cell lysates harvested from an equivalent number of cells for ATPaseTb2 RNAi non-induced and induced cells throughout the RNAi time course (Fig. 4B). Western blot analysis using the antibody against ATPaseTb2 exhibited a reduction of the targeted protein by 73% at day 2 of RNAi induction. A non-specific band detected on the same membrane was used to determine equal loading of the samples.

With the efficient knockdown of ATPaseTb2 verified, an *in vivo* assay was employed to measure the Δψ_m_ in the ATPaseTb2 KD cell population (Fig. 4C), since the Δψ_m_ is generated by the F_o_F_1_-ATPase in BF *T*. *brucei* [9]. Flow cytometry analysis was used to determine the changes observed in these cells stained with Mitotracker Red CMX-Ros, whose fluorescent intensity is proportionally dependent on the strength of the Δψ_m_. A substantial decrease of the Δψ_m_ in the KD cell population was observed two days (IND2) after RNAi induction, coinciding with the first time point to display a significant growth inhibition. Importantly, this result confirms the vital function of ATPaseTb2 within the F_o_F_1_-ATPase.

### Depleting ATPaseTb2 does not significantly alter ATP hydrolysis capabilities

**Fig 5:**
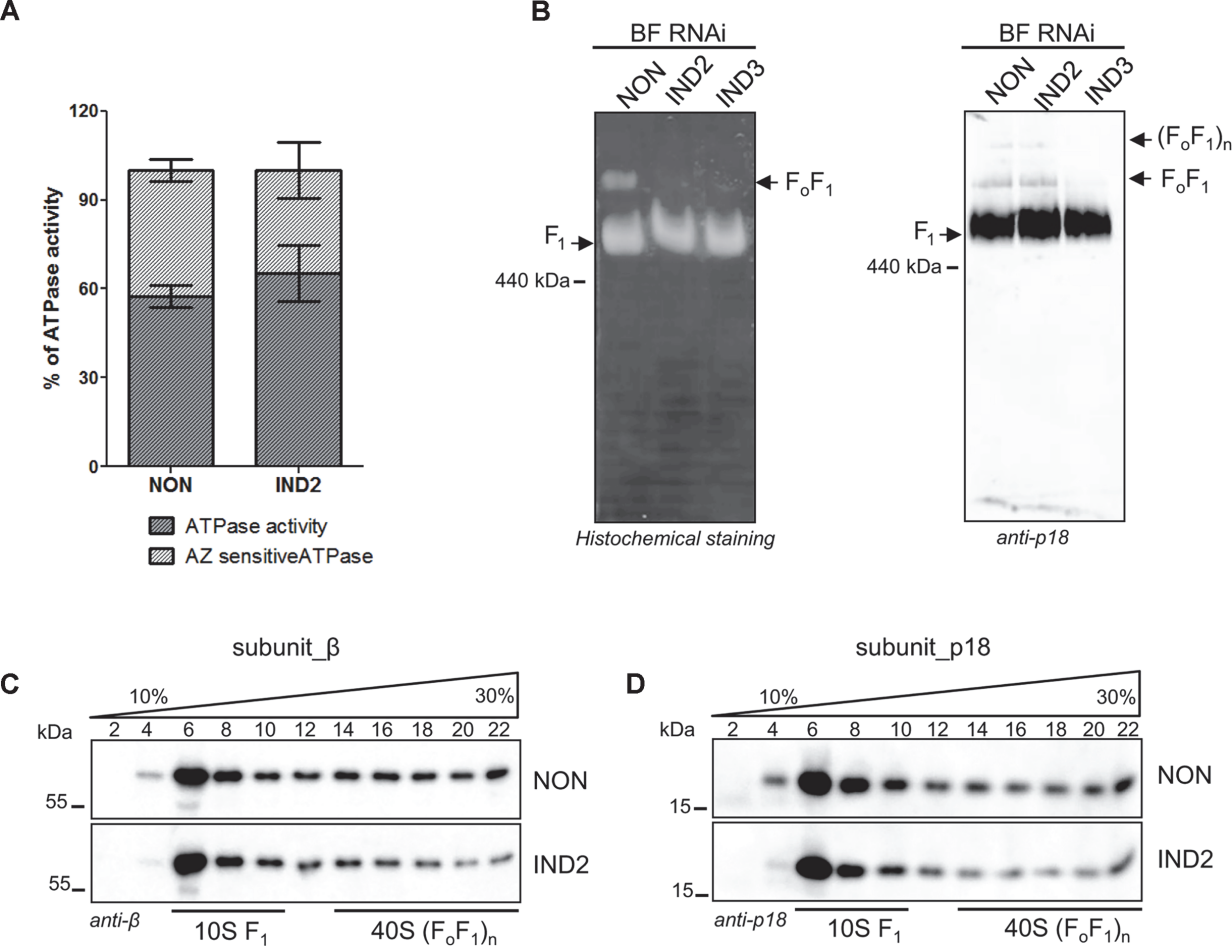
ATPaseTb2 depletion does not appreciably affect F_1_-ATPase activity in BF *T*. ***brucei* cells, but it does significantly diminish the stability of F**_**o**_ **F**_**1**_ **-ATPase complexes**. A) Employing the Sumner ATPase assay, the F_1_-ATPase hydrolytic activity was measured in ATPaseTb2 RNAi cells either not induced (NON) or induced for 2 days (IND2). Crude mt vesicles were obtained by digitonin extraction and the ATPase activity was assayed by measuring the release of free phosphates. The specific F_1_-ATPase inhibitor, azide (AZ, 2 mM), was added as indicated. The total amount of free-phosphate created from all ATPase enzymes present in the sample was set at 100% (hatched column). The azide-sensitive activity representing the F_1_-ATPase is depicted in dark grey. The results are means ± s.d. (n = 4). B) In-gel ATP hydrolysis activity of F_o_F_1_-ATPase was visualized after ATPaseTb2 reduction. Mitochondria from RNAi non-induced cells (NON) and cells induced for 2 (IND2) and 3 (IND3) days were lysed with 2% dodecyl maltoside. Equal amounts of lysed mitochondrial proteins (100 μg) were fractionated on a 2%-12% BNE and the F_o_F_1_-ATPase activity was visualized by in-gel histochemical staining resulting in a white lead phosphate precipitate. Positions of F_1_-ATPase and monomeric F_o_F_1_-ATPase are depicted. The size of equine spleen ferritin (440 kDa) is indicated. C) The stability of F_o_F_1_-ATPase complexes upon ATPaseTb2 silencing was examined using hrCNE. Mitochondria from RNAi non-induced cells (NON) and cells induced for 2 (IND2) and 3 (IND3) days were lysed by digitonin (4 mg/mg). Equal amounts of lysed mitochondrial proteins (20 μg) were fractionated on a 3%-12% hrCNE, blotted onto a nitrocellulose membrane and probed with the anti-p18 antibody. Positions of F_1_-ATPase and monomeric and dimeric F_o_F_1_-ATPases are depicted by arrows. The size of ferritin from equine spleen (440 kDa) is indicated. D) The sedimentation profile of F_o_F_1_-ATPase complexes was examined using western blot analysis of glycerol gradient fractions. Mitochondria from RNAi non-induced cells (NON) and cells induced for 2 days (IND2) were lysed with 1% Triton X-100. An equal amount of the cleared lysates (3,3 mg) were loaded on a manually poured 10–30% glycerol gradient. Western analyzes with anti-β and anti-p18 antibodies depicted the sedimentation profile of the F_o_F_1_-ATPase complexes. The manually fractionated glycerol gradient fractions are labelled and sizes of the protein marker are indicated.

To assess if the decrease in the Δψ_m_ is the result of an impaired ATP hydrolytic activity of the enzyme, the total ATPase activity was measured in mt lysates with or without azide, an inhibitor of the catalytic F_1_-ATPase [43]. Typically, the specific F_1_-ATPase activity represents ∼ 35–45% of total mt ATPase activity. Our results indicate that neither the total ATPase nor azide-sensitive activities were significantly altered between the non-induced and ATPaseTb2 RNAi-induced cells (Fig. 5A). ATPase activity can also be visualized by in-gel activity staining when mitochondria from these cells are solubilized with dodecyl maltoside and separated on a 2–12% blue native PAGE (BNE) [41]. The specific ATPase staining revealed two major bands in non-induced cells, the predominant lower one representing the activity produced from the F_1_-moiety and the less significant higher band representing the hydrolytic activity of the monomeric F_o_F_1_-ATPase (Fig. 5B). It is important to note that solubilizing mitochondria with any detergent can result in unintended structural consequences not found under normal physiological conditions, especially for a large complex comprised of a matrix moiety and a membrane embedded region. Although detergents tend to dissociate the F_1_-moiety from F_o_F_1_-ATPase complexes, it is intriguing that there is still a portion of ATP hydrolysis being produced from the complete F_o_F_1_-ATPase in the non-induced BF cells. Conspicuously, this activity is absent when ATPaseTb2 RNAi is induced (Fig. 5B).

### Loss of ATPaseTb2 disrupts the F_o_F_1_-ATPase complexes in BF *T*. *brucei*

The absence of activity for the F_o_F_1_-monomeric complex might be explained by complex instability. Thus, the structural integrity of the coupled F_o_F_1_-complex was further analyzed using hrCNE, which were loaded with digitonin-lysed mitochondria from non-induced and ATPaseTb2 RNAi induced cells. After transferring the separated native protein complexes to a nitrocellulose membrane, a specific antibody against the p18 subunit detected the F_1_-moiety as well as both monomeric and oligomeric F_o_F_1_-complexes (Fig. 5C). Interestingly, we repeatedly observed that the abundance of the monomeric and oligomeric complexes is decreased after RNAi induction and by day 3 these complexes are not detected at all. These results were complemented by the sedimentation profile of F_o_F_1_-ATPase complexes on glycerol gradients. Mitochondria were isolated from non-induced RNAi cells and cells induced for two days (IND2). These were then lysed by 1% Triton X-100 and an equal amount of mt protein from each sample was fractionated on a 10–30% GG. The resolved fractions were then analyzed by western blot and the anti-β and anti-p18 antibodies demonstrated the effect of the ATPaseTb2 KD on the F_o_F_1_-ATPase sedimentation profile. As indicated in Fig. 5D and the corresponding scanning densitometry results listed in S2 Fig., two days of RNAi induction resulted in a diminished amount of β and p18 signals from the higher S-values (IND2 panels), while the GG sedimentation pattern of RNAi non-induced cells was more similar to wild type BF427 (see Fig. 3C). These results are in agreement with the PF RNAi ATPaseTb2 cell lines, in which the stability of the monomeric and multimeric F_o_F_1_-ATP synthases was significantly affected upon RNAi [26].

Using a variety of methods employing various detergent types, we have established that the ATPaseTb2 subunit is an essential component of the F_o_F_1_-ATPase monomer and higher oligomers in the bloodstream stage of *T*. *brucei*. Furthermore, the depletion of ATPaseTb2 diminishes the abundance of membrane-bound F_o_F_1_-ATPase complexes, which directly initiates a substantial decrease in the Δψ_m_ that manifests as a strong growth phenotype.

### ATPaseTb2 depletion affects cell growth, the Δψ_m_ and F_o_F_1_-ATPase integrity in Dk *T*. *b*. *evansi*

**Fig 6:**
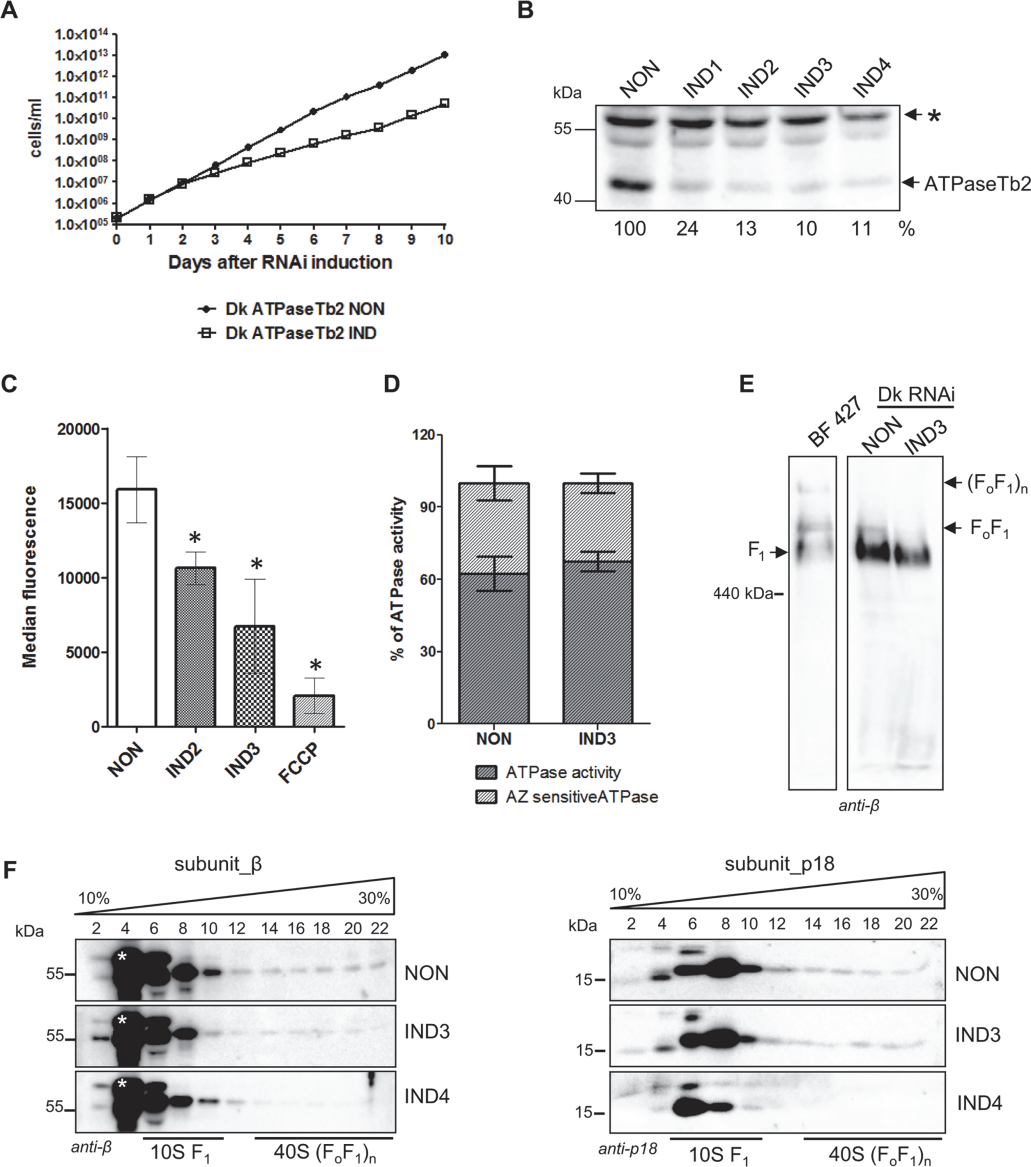
Cell growth, Δψ_m_ maintenance and the stability of FoF1-ATPase complexes are all affected by the loss of ATPaseTb2 in dyskinetoplastic ***T.b. evansi***. A) Growth curves of the non-induced (NON) and induced (IND) ATPaseTb2 RNAi Dk *T*. *b*. *evansi* cell lines were measured for 10 days. Cells were maintained in the exponential growth phase (between 10^5^ and 10^6^ cells/ml) and the cumulative cell number represents the cell density adjusted by the daily dilution factor. The figure is representative of three independent RNAi-inductions. B) The steady-state abundance of ATPaseTb2 in non-induced (NON) cells and cells harvested 1, 2, 3 and 4 days post RNAi induction (IND1, IND2, IND3, IND4) was determined by western blot analysis using a specific ATPaseTb2 antibody. Mt Hsp70 served as a loading control. The numbers underneath the ATPaseTb2 panel represent the abundance of immunodetected protein expressed as a percentage of the non-induced samples after normalizing to the loading control. The pertinent sizes of the protein marker are indicated on the left. The figure is a representative western blot from three independent RNAi-inductions. C) The Δψ_m_ was measured in non-induced (NON) ATPaseTb2 RNAi cells and cells induced for 2 and 3 days (IND2 and IND3) by flow cytometry using Mitotracker Red CMX-Ros. The results are means ± s.d. (n = 3). *P< 0.05, Student’s *t*-test. D) The F_1_-ATPase hydrolytic activity was measured for induced (IND3) or non-induced Dk ATPaseTb2 RNAi cells as described in Fig. 5A. The results are means ± s.d. (n = 4). E) The stability of F_o_F_1_-ATPase complexes upon ATPaseTb2 depletion was examined using hrCNE. Mitochondria from wild type BF427, ATPaseTb2 RNAi *T*. *b*. *evansi* non-induced cells (NON) and cells induced for 3 days (IND3) were lysed by digitonin (4 mg/mg). Equal amounts of lysed mitochondrial proteins (20 μg) were fractionated on a 3%-12% hrCNE, blotted onto a nitrocellulose membrane and probed with an anti-β antibody. Positions of F_1_-ATPase and monomeric and multimeric F_o_F_1_-ATPases are depicted by arrows. The size of the equine spleen ferritin (440 kDa) is indicated on the left. F) The sedimentation profile of F_o_F_1_-ATPase complexes was examined using western blot analysis of glycerol gradient fractions. Mitochondria from RNAi non-induced cells (NON) and cells induced for 3 and 4 days (IND3 and IND4) were lysed by 1% Triton X-100 and an equal amount of the cleared samples (1.3 mg) were loaded on a 10–30% glycerol gradient. Western analyzes with anti-β and anti-p18 antibodies depicted the sedimentation profile of the F_o_F_1_-ATPase complexes. The glycerol gradient fractions are labelled and the relevant sizes of the protein marker are indicated. A nonspecific band only visible in Dk164 gradients is indicated by an asterisk.

To further dissect the primary role of the membrane-bound ATPaseTb2 subunit, we investigated the effect of targeted gene silencing in transgenic Dk *T*. *b*. *evansi*, which facilitate the tet inducible expression of dsRNA. These Dk cells were transfected with an ATPaseTb2 RNAi vector and several positive clones were screened for a growth phenotype. As shown in Fig. 6A, the surprising inhibition of cell growth in RNAi induced cells appeared two days after tet induction and the doubling time remained reduced throughout the whole ten day experiment. Furthermore, western analysis of whole cell lysates harvested over the RNAi time course exhibited a significant reduction of ATPaseTb2 in the RNAi induced cells beginning on day 1 (Fig. 6B), while the analysis of a non-specific band served as the loading control. Interestingly, if the only role of the subunit was to stabilize the proton pore, it presumably would not be essential in cells that lack mtDNA and have adapted to the loss of a functioning proton pore. However, these results suggest that ATPaseTb2 is important for F_o_F_1_-ATPase complexes in these unique cells and thus this cell line was further exploited to characterize the function of ATPaseTb2.

Since the Δψ_m_ in Dk cells is generated using the hydrolytic activity of the F_1_-moiety coupled with ATP/ADP translocation, the Δψ_m_ was examined in ATPaseTb2 non-induced and RNAi induced cell populations. As shown in Fig. 6C, a reduction of the Δψ_m_ was detected in the RNAi induced cell population after 2 (IND2) and 3 days (IND3). Since the reduction of the Δψ_m_ already appears at day 2 of tet induction and the growth phenotype does not truly manifest until day 3, we postulate that the observed decrease of the Δψ_m_ is a direct effect of ATPaseTb2 silencing and not a consequence of decreased cell viability. Furthermore, to assess if this decreased Δψ_m_ is caused by an impairment of the F_1-_ATPase hydrolytic activity, the total ATPase activity was measured in mt lysates with or without azide. Similar to BF RNAi ATPaseTb2 cells (Fig. 5A), no changes in total or azide-sensitive activities were observed between the non-induced and ATPaseTb2 RNAi-induced cells (Fig. 6D).

The structural integrity of Dk F_o_F_1_-ATPase complexes was then investigated using mild non-denaturing hrCNE to fractionate mitochondrial proteins purified from BF427 cells and Dk cells either induced for ATPaseTb2 RNAi or not. A western blot containing BF427 mitochondria as a reference sample was probed with an anti-β antibody and used as marker to visualize each state of the F_1_-moiety, either isolated by itself or being partnered with the monomeric and multimeric F_o_F_1_-complexes. Strikingly, while a majority of the β subunit signal in Dk RNAi non-induced cells was observed in the region of isolated F_1_-ATPase, we also consistently detected an irrefutable signal corresponding to the F_o_F_1_-monomeric complex, presumably assembled intact with the exception of subunit a (Fig. 6E). While there is evidence of mammalian rho cells containing a similar complex [44–46], this is the first time it has been demonstrated in the comparable Dk trypanosomatids. Furthermore, the induction of ATPaseTb2 RNAi in the Dk cells led to the disappearance of the monomeric complex, leaving only the F_1_-ATPase to be detected on the western blot (Fig. 6E). Once again, the evidence indicates that ATPaseTb2 is important for the structural integrity of the F_o_F_1_-ATPase and its depletion in Dk *T*.*b*. *evansi* cells leads to the disruption of the unique membrane-bound F_o_F_1_-ATPase complexes.

To corroborate this observation, we purified mitochondria from Dk *T*. *b*. *evansi* non-induced and ATPaseTb2 RNAi-induced cells and lysed them with Triton X-100. These gently disrupted lysates were then fractionated on glycerol gradients, which revealed that a majority of the signal from subunits β and p18 was located within fractions 6–10, representing the F_1_-moiety (Fig. 6F). Nevertheless, weaker bands representing the F_o_F_1_-ATPase complexes (fractions 14–22) were again detected in the non-induced samples (Fig. 6F NON panels). Noticeably, these same protein markers in the mt fractionation of ATPaseTb2 RNAi induced cells displayed a significant reduction in fractions 14–22 (Fig. 6F IND4 panels), where the F_o_F_1_- monomer/oligomer sediments. This observation is quite surprising since the main role of this enzyme is to create the Δψ_m_, presumably using only the hydrolytic activity of the matrix facing F_1_-moiety. In an attempt to determine if a portion of the F_1_-ATPase is membrane associated and that the disruption of this attachment is the reason for the observed decrease in the Δψ_m_ and the detected growth phenotype, we decided to visualize these complexes *in situ* using immunogold labelling followed by electron microscopy.

### Loss of ATPaseTb2 leads to the dissociation of F_1_-ATPase from the mt membrane

Immunogold labeling with a primary anti-β antibody was performed on ultrasections of Dk *T*. *b*. *evansi* non-induced (NON) and ATPaseTb2 RNAi induced cells for 3 (IND3) and 5 (IND5) days. Electron micrographs illustrate that Dk trypanosomes have a simple and reduced mitochondrion (S3A Fig.), lacking both cristae and a typical kinetoplast structure, as previously reported for the dyskinetoplastic *T*. *b*. *evansi* [17]. Whenever these reduced double-membrane organelles could be unequivocally identified, we tallied the number of immunogold beads either in the close proximity of the mt membrane or in the matrix (S3A Fig.). Strikingly, 70% of the gold particles identified were found associated with the mt membrane in the NON images (S3B Fig.). From the 113 images captured from either NON, IND3 or IND5 samples, we performed a Chi-squared analysis on the immunogold beads associated with the mt membrane to determine if their distribution is random (S3C Fig.). With two degrees of freedom, the calculated χ^2^ = 43.5 (p < 0.0001), which signifies that the difference between the observed and expected particles was statistically significant. These values were then plotted to determine the relative labelling index (RLI = N_obs_/ N_exp_) for the mt membrane associated gold particles (S3D Fig.). This analysis reveals that the observed number of gold particles found in close proximity to the mt membrane for the NON electron micrographs is significantly greater than if the particles were randomly distributed (RLI = 1), while the opposite is true for the IND5 samples. Since we understand the general subjectivity of this method, we performed this experiment in a blinded study and only considered a gold bead to be associated with the mt membrane if it was in the immediate proximity of the membrane. For the very few scored beads that could potentially fall in a membrane-bound grey zone, it should be noted that the physical distance from the subunit β antigen to the gold particle can be up to 15–20 nm, not to mention that subunit β itself is projected from the membrane by subunit γ by as much as 13 nm [47,48]. Furthermore, very few immunogold beads were detected outside of defined mt structures and each mt image only contained 1–8 immunogold beads (average of 2.4 beads/mt image), suggesting that the labelling was very specific for our abundant mt antigen. It is important to note, that utilizing an assay that does not require the use of any detergents, we demonstrated that a majority of the F_1_-ATPase is located within close proximity of the mt membrane and this association is disrupted when ATPaseTb2 is depleted.

## Discussion

The *T*. *brucei* F_o_F_1_-ATPase has obtained unique properties in comparison to its eukaryotic counterparts. The occurrence of novel subunits combined with the lack of typical eukaryotic subunits that compose the F_o_ membrane-bound moiety and the peripheral stalk is intriguing from an evolutionary viewpoint. Because these atypical subunits lack significant homology to known proteins, questions arise concerning their authenticity as genuine subunits of the complex and what their function and localization within the complex might be. Here, we focused on the functional characterization of one of the trypanosomatid specific subunits, ATPaseTb2 in the bloodstream stages of *T*. *brucei*. Notably, we determined that its function is essential for maintaining the normal growth rate of the infectious stage of *T*. *brucei* and also for the important veterinary dyskinetoplastic parasite, *T*. *b*. *evansi*. Additional analyses demonstrated that the ATPaseTb2 is membrane embedded and a component of the F_o_F_1_-ATPase that is present in both BF and Dk cells. Furthermore, the depletion of this F_o_-moiety subunit results in a decreased Δψ_m_ and a loss of F_o_F_1_-ATPase complexes. Combined with bioinformatic tools that predict a transmembrane domain and identify a low homology to subunit d, we suggest that ATPaseTb2 might be a component of the peripheral stalk of the F_o_F_1_-ATPase.

The peripheral stalk is indispensable for ATP synthesis as it serves to impede the movement of the catalytic α_3_β_3_ headpiece while the proton motive force rotates the c-ring and the connected asymmetrical central stalk in a way that imposes conformational changes in the catalytic nucleotide binding sites of the β subunits. The peripheral stator is also essential when the complex harnesses the energy provided from ATP hydrolysis to drive the rotation of the enzyme in reverse, allowing protons to be pumped across the mt inner membrane to produce the Δψ_m_ when physiological conditions dictate.

The bovine and yeast peripheral stalk is composed of four subunits, OSCP, b, d, and F_6_/h (bovine/yeast nomenclature). Subunit b (∼20kDa protein) contains two trans-membrane segments at the N-terminus, while the rest of the protein is hydrophilic—protruding into the matrix and interacting with OSCP and subunit d [23,49–51]. The interaction between subunit b and OSCP is stabilized by subunit F_6_ [22]. The predominantly hydrophilic subunit d interacts with all three mentioned subunits and it has been shown to be essential for the function of the F_o_F_1_-complex. The yeast knock-out of subunit d was characterized by the de-attachment of the catalytic F_1_-ATPase from the protonophoric sector, the loss of detectable oligomycin sensitive ATPase activity and the absence of subunit a in the F_o_-moiety [36]. Notably, of these four subunits, the only homolog identified in the *T*. *brucei* genome is OSCP and its protein product was identified as a bona fide subunit of the purified F_o_F_1_-ATP synthase [26]. Homologs for subunits b, d, and F_6_ are missing and most likely have been replaced by the novel proteins that associate with the *T*. *brucei* F_o_F_1_-ATP synthase. Here we demonstrated that the ATPaseTb2 subunit is a membrane-bound protein, containing one predicted transmembrane domain, with a large hydrophilic region extending into the matrix that possesses a low homology to the bovine subunit d. In accordance with other yeast or bovine subunits of the peripheral stalk, the down-regulation of ATPaseTb2 affects the stability of the F_o_F_1_-complex and its oligomycin sensitivity (this study, [26]).

Considering the relatively large molecular mass of ATPaseTb2 (43 kDa), it is plausible to speculate that ATPaseTb2 functionally represents the membrane-bound subunit of the peripheral stalk fused with subunit d, offering an early attempt by eukaryotes to add layers of complexity that will allow for greater adaptability. It is noteworthy to mention that a species-specific architectural variant of the peripheral stalk was also proposed for colorless and green algae, where the novel subunits Asa2, Asa4 and Asa7 fulfil a structural role in forming the peripheral stalk [52,53]. Other discrepancies from the typical eukaryotic enzyme model can be found in the ciliate *Tetrahymena thermophila*, representing the superphylum of Alveolates, where the homolog of the conserved subunit b has so far not been identified in the genome. Instead, three novel proteins detected in this ATP synthase complex have been proposed to substitute for subunit b [28]. Thus, it seems that the composition and overall structural appearance of the F_o_F_1_-ATP synthase from organisms representing different lineages other than Opisthokonts, might be more diverse than previously thought and deserve more attention from basic researchers to elucidate alternate evolutionary solutions to a common thread of life.

**Fig 7:**
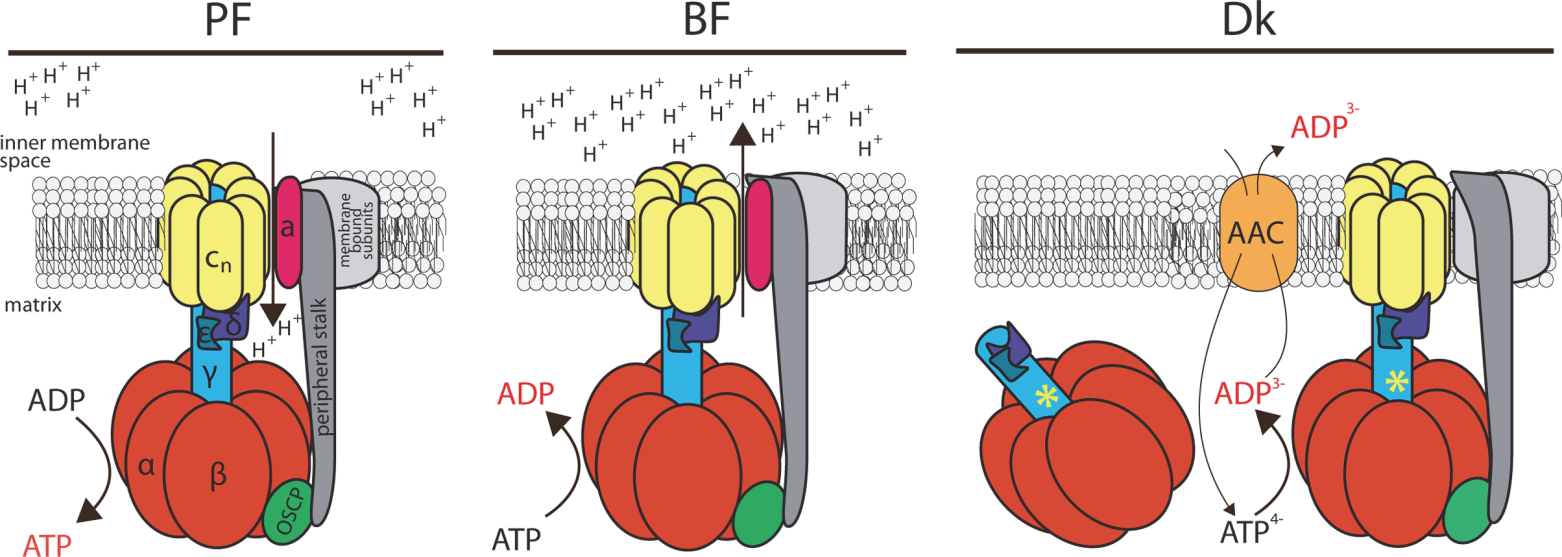
Schematic representation of the F_o_F_1_-ATP synthase/ATPase complex in trypanosome mitochondria. The F_o_F_1_-ATP synthase possesses the conventional function in the procyclic form (PF) of the parasite, coupling transmembrane proton transfer with ATP synthesis. In contrast, this enzymatic complex works in reverse in the bloodstream form (BF) of the parasite, hydrolyzing ATP to generate the Δψ_m_ in the absence of canonical cytochrome-containing respiratory complexes. This hydrolytic activity is also utilized in the dyskinetoplastid (Dk) form, which lacks mitochondrial DNA and thus a proton pore. Therefore, in Dk cells, enhanced ATP hydrolysis by a mutated F_1_-moiety (a necessary mutation in the γ subunit is marked with the asterisk) provides substrate for the ATP/ADP carrier (AAC) and the Δψ_m_ is produced by the electrogenic exchange of ADP^3-^ for ATP^4-^. Furthermore, we present that a minor fraction of F_1_-ATPase still appears to be associated with F_o_ and that association may be functionally important for maintaining the Δψ_m_. Orthologues of the colored subunits (α, β, γ, δ, ε, OSCP, a and c) have been annotated in trypanosomes. In addition to OSCP, the peripheral stalk (dark grey) is usually composed of subunits b, F_6_ and d, but these homologs have not been identified in the *T*. *brucei* genome. Also, conspicuously absent are subunits A6L, e, f and g, which are all membrane-bound (light grey).

*T*. *brucei* is a member of the Excavata clade and represents a desirable model to study the function and regulation of the F_o_F_1_-ATP synthase/ase complex since it utilizes the proton motive force to produce ATP in the PF life stage, while in the BF stage it hydrolyzes ATP to pump the protons necessary to create the Δψ_m_. Moreover, dyskinetoplastic *T*. *b*. *evansi* employs the hydrolytic activity of the F_1_-ATPase and the electrogenic exchange of ADP^3-^ for ATP^4-^ by the AAC as yet another strategy to generate the Δψ_m_ (Fig. 7). Therefore, it was surprising that the ATPaseTb2 protein was detected in all four examined cell types (PF, BF, Dk 164 and *T*. *b*. *evansi*), albeit at a much lower abundance in BF and Dk cells, but that is in full agreement with the reduced mitochondria previously defined for these two forms [39]. Furthermore, when PF and BF mt lysates were resolved by GG and hrCNE, ATPaseTb2 was only detected when co-localized with the monomeric and oligomeric F_o_F_1_-ATP synthase/ase complexes. Interestingly, similar results were obtained from the mt lysates of cells lacking mtDNA. Considering that only the F_1_-ATPase was previously assumed to be important for maintaining the Δψ_m_ in the Dk trypanosomes, the presence of these complexes is intriguing. Nevertheless, a proteomics study performed with osteosarcoma 143B ρ° cells revealed that in addition to the F_1_-ATPase subunits, subunit d of the peripheral stalk was also identified [54]. In a similar project involving fibroblast MRC5 ρ° cells, the oligomycin insensitive complex was purified and determined to contain several F_o_ (b and c) subunits along with a couple of peripheral stalk subunits (OSCP, d). Notably, this complex was loosely associated with the mt membrane [45]. Furthermore, several additional studies have shown that the F_1_-ATPase in yeast or mammalian cells lacking a mt genome (i.e. mammalian subunits a/ATP6, A6L; yeast subunits 6, 8 and 9) is membrane associated, with the attachment most likely occurring via the central and/or peripheral stalk [44,46,55,56]. Our data also suggest that since ATPaseTb2 membrane-bound complexes (monomeric and multimeric) do not display significant differences in their native size or sedimentation values, then the mt encoded subunit a might be the only missing subunit from these complexes. Such a uniquely structured complex can be explained by the current model for the assembly of the F_o_F_1_-ATPase, in which the F_o_ subunit a is the last protein incorporated into the enzyme to ensure that it is able to function properly before introducing a complete proton pore. Furthermore, this integration is dependent on the presence of subunit b of the peripheral stalk [57].

RNAi silencing of ATPaseTb2 in BF cells caused a strong growth phenotype concurring with a decreased Δψ_m_. Importantly, ATPaseTb2 also proved to be essential for maintaining the normal growth rate and the Δψ_m_ of Dk cells. Combined with the hrCNE and glycerol gradient sedimentation assays that revealed a decrease of the high molecular weight complexes, but not the F_1_-ATPase, our studies suggest that these membrane-bound enzymes might significantly contribute to the membrane potential in Dk cells. The biological significance of this localization of the F_1_-ATPase might be to efficiently coordinate the activity of this enzyme with its substrate transporter, both of which are responsible for producing the Δψ_m_ in Dk. By restricting the F_1_-ATPase to the mt membrane, it keeps it within the close vicinity of its biochemically functional partner, the AAC. In this way, it helps ensure that the hydrolysis of ATP results in a relatively concentrated region of substrate for the mt membrane embedded AAC, especially in a mitochondrion lacking defined cristae and the microenvironments they create. Indeed, there is precedence for such a close spatial collaboration to increase efficiency, as an actual physical interaction between these two enzymes has been reported as the ATP synthasome in mammals [40,58]. We are currently building a set of tools to perform additional functional assays and imaging techniques that we hope will further resolve the function of the F_o_F_1_-ATPase complexes in these pathogenic trypanosomes.

## Materials and Methods

### Plasmid construction

The ATPaseTb2 (Tb927.5.2930) RNAi constructs targeted an 825 bp fragment of the gene that was PCR amplified from *T*. *brucei* strain 427 genomic DNA with the following oligonucleotides: FW—CACGGATCCATGCGCCGTGTATC, REV-CACCTCGAGTTCGGCCCGATC. Utilizing the BamHI and XhoI restriction sites inherent in the primers (underlined), this fragment was cloned into the p2T7-TA blue plasmid [59] (used to create a BF RNAi cell line) and the p2T7–177 plasmid [60] (used to generate a Dk RNAi cell line). For the inducible expression of ATPaseTb2 fused with a C-terminal 3xv5 tag, the ATPaseTb2 coding sequence was PCR amplified (Fw: ACAAAGCTTATGC GCCGTGTATC, Rev: ATAGGATCCGTGATGGGCCTTTTTC) and cloned into the pT7_v5 vector using HindIII and BamHI restriction enzymes [61]. The pLEW79MHTAP vectors for the tet inducible expression of ATPaseTb2 and Tb927.5.1710 (F_o_F_1_-ATPase subunit p18) TAP-tagged proteins were previously described in [26].

### *Trypanosoma* culture conditions and generation of cell lines

Bloodstream form *T*. *brucei brucei* Lister 427 strain, stable acriflavin-induced dyskinetoplastic *T*. *b*. *brucei* EATRO 164 [18] and dyskinetoplastic *T*. *b*. *evansi* Antat 3/3 [62] were all grown *in vitro* at 37°C and 5% CO_2_ in HMI-9 media containing 10% FBS. The BF427 single marker cell line [63] and a *T*. *b*. *evansi* cell line [14], both constitutively expressing the ectopic T7 RNA polymerase and tet repressor (TetR were used for the tet inducible expression of dsRNA and the TAP- and v5-tagged proteins. Each plasmid was linearized with NotI enzyme and transfected into the appropriate cell line as described previously [63]. Linearized p2T7–177 was targeted to the minichromosome 177 basepair repeat region, while linear pTv5 and pLEW79MHTAP vectors were integrated into the rDNA intergenic spacer region [60,61,63]. The induction of RNAi and ectopically expressed tagged proteins was triggered by the addition of 1 μg/ml of tet into the media. Cell densities were measured using the Z2 Cell Counter (Beckman Coulter Inc.). Throughout the analyses, cells were maintained in the exponential mid-log growth phase (between 1x10^5^ to 1x10^6^ cells/ml).

### Tandem affinity purification (TAP) of tagged complexes

The TAP protocol was generously provided by L. Jeacock and A. Schnaufer (personal communication) and optimized for BF cells. Briefly, 2x10^8^ cells were harvested and lysed for 20 min on ice with 1% Triton X-100 in an IPP150 buffer (150 mM NaCl, 0,1% NP40, 10mM Tris-HCl pH 8.0) containing Complete protease inhibitors (Roche). The lysates were then cleared by centrifugation (16,000 g, 15 min, 4°C). Meanwhile, IgG antibodies were covalently bound to Dynabeads M-270 Epoxy (Invitrogen) using a protocol previously described [64]. Charged beads were blocked with 1% BSA and then incubated with the cleared cell lysates (4°C for 2 hours, constantly rotating). The beads were then washed three times with IPP150 and once with a TEVCB buffer (150 mM NaCl, 0.1% NP40, 0.5 mM EDTA, 1 mM DTT, 10 mM Tris-HCl, pH 8.0). Finally, the bound protein complexes were released by AcTEV protease (Invitrogen) cleavage and the eluate was analyzed by SDS-PAGE.

### Isolation of mt vesicles

Crude mt vesicles were obtained by hypotonic lysis as described in detail earlier [65]. Briefly, cell pellets from ∼2x10^9^ cells were washed with SBG (150 mM NaCl, 20 mM glucose, 20 mM NaHPO_4_, pH 7.9), resuspended in DTE (1 mM Tris, 1 mM EDTA, pH 8.0) and homogenized in a Dounce homogenizer. Alternatively, for a smaller scale hypotonic isolation, cell pellets from ∼1x10^9^ cells were washed in NET buffer (150 mM NaCl, 100 mM EDTA, 10 mM Tris, pH 8.0), resuspended in DTE and homogenized by passing through a 25G needle. To re-create the physiological isotonic environment, 60% sucrose was immediately added to the lysed cells to reach a final concentration of 250 mM. Samples were spun down at 15,000 g, 10 min, 4°C to clear the soluble cytoplasmic material from the lysates. The resulting pellets were resuspended in STM (250 mM sucrose, 20 mM Tris pH 8.0, 2 mM MgCl_2_), supplemented with a final concentration of 3 mM MgCl_2_ and 0.3 mM CaCl_2_ before incubating with 5 μg/ml DNase I for 1hr on ice. Then an equal volume of STE buffer (250 mM sucrose, 20 mM Tris pH 8.0, 2 mM EDTA pH 8.0) was added and the material was centrifuged at 15,000 g, 10 min, 4°C. Pellets enriched with the mt membrane vesicles were washed in STE and kept at -70°C.

### SDS-PAGE and Western blot

Protein samples were separated on SDS-PAGE, blotted onto a PVDF membrane (PALL) and probed with the appropriate monoclonal (mAb) or polyclonal (pAb) antibody. This was followed by incubation with a secondary HRP-conjugated anti-rabbit or anti-mouse antibody (1:2000, BioRad). Proteins were visualized using the Pierce ECL system (Genetica/Biorad) on a ChemiDoc instrument (BioRad). When needed, membranes were stripped at 50°C for 30 min in a stripping buffer (62.5 mM Tris pH 6.8, 100 mM mercapthoethanol, 2% SDS) and re-probed. The PageRuler prestained protein standard (Fermentas) was used to determine the size of detected bands. Primary antibodies used in this study were: mAb anti-v5 epitope tag (1:2000, Invitrogen), mAb anti-mtHsp70 (1:2000) [66], pAb anti-AAC (1:2000) [67], pAb anti-MRP1 (1:1000) [68], pAb anti-APRT (1:1000) and pAb anti-enolase (1:1000) [69]. Antibodies against ATPaseTb2 (1:1000), ATPaseTb1 (1:1000), subunit β (1:2000) and subunit p18 (1:1000) were prepared for the purpose of this study. The open reading frames of the respective genes without their predicted mt localization signal were cloned into the *E*. *coli* expression plasmid, pSKB3. The proteins were overexpressed in BL21 *E*. *coli* cells and purified under native conditions (subunit β) or denatured conditions (ATPaseTb2, ATPaseTb1 and p18) using a 6 M guanidinium lysis buffer and 8 M urea binding buffer. The denatured proteins were then refolded with a step-wise dialysis procedure that included 0.5 M arginine in the refolding buffer. Native and refolded antigens were sent to Pineda (Antikörper-Service, Germany) or Davids Biotechnologie (Regensburg, Germany) for polyclonal antibody production.

### Mt membrane potential (Δψ_m_) measurement

The Δψ_m_ was determined utilizing the red-fluorescent stain Mitotracker Red CMX-Ros (Invitrogen). Cells in the exponential growth phase were stained with 100 nM of the dye for 30 min at 37°C. Cells were pelleted (1,300 g, 10 min, RT), resuspended in 2 ml of PBS (pH 7.4) and immediately analyzed by flow cytometry (BD FACS Canto II Instrument). For each sample, 10,000 events were collected. Treatment with the protonophore FCCP (20 μM) was used as a control for mt membrane depolarization. Data were evaluated using BD FACSDiva (BD Company) software.

### F_1_- ATPase assay

ATPase activity was measured with the Sumner assay, which is based on the release of free phosphate when ATP is hydrolyzed by the enzyme as described earlier [14,70]. Briefly, crude mt lysates were obtained from 2x10^8^cells by SoTe/digitonin extraction (0.015% digitonin, 0.6 M Sorbitol, 2 mM EDTA, 20 mM Tris-HCl pH 7.5). Mt pellets were resuspended in an assay buffer (200 mM KCl, 2 mM MgCl_2_, Tris-HCl pH 8.0) and the 20 min reaction was initiated by the addition of ATP to a final concentration of 5 mM. Where indicated, samples were pre-treated with the F_1_-ATPase specific inhibitor, sodium azide (2 mM) for 10 min at 37°C. The 100 μl enzymatic reactions were deproteinated by the addition of 1.9 μl of 70% perchloric acid. After a 30 min incubation on ice, the samples were spun down (16,000g, 10 min, 4°C) and 90 μl of the supernatant was incubated for 10 min with 0,5 ml of Sumner reagent (8.8% FeSO_4_.7H_2_O, 375 mM H_2_SO_4_, 6.6% (NH_4_)Mo_7_O_24_.4H_2_O)[62]. 200 μl was then transferred to a 96 well plate and the absorbance was measured at 610 nm using a Tecan Infinite plate reader (Infinite M200Pro, Tecan). To calibrate the assay, a standard curve was calculated from the absorbance values of linear inorganic phosphate samples (0–2 mM).

### In-gel histochemical staining of F_1_-ATPase activity

Blue native PAGE (BNE) of mt lysates, followed by in-gel activity staining was adapted from published protocols [26,32]. Briefly, mt vesicles from ∼2x10^9^ cells were resuspended in a mt lysis buffer (0,75 M amino-n-caproic acid—ACA, 50 mM Bis-Tris, 0,5 mM EDTA, pH 7.0), lysed with 2% dodecylmaltoside (DDM) for 1hr on ice and then cleared by centrifugation (16,000g for 30 min, 4°C). The protein concentration of each mt lysate was determined by a Bradford assay (BioRad), so that 100 μg of total mt protein could be mixed with 1 M ACA and 5% Coomassie brilliant blue G-250 before being loaded on a 2–12% Bis-Tris BNE gel. Immediately after the run (3 hr, 100 V, 4°C), the gel was incubated overnight in an ATPase reaction buffer (35 mM Tris pH 8.0, 270 mM glycine, 19mM MgSO4, 0.3% Pb(NO_3_)_2_, 11 mM ATP).

### High resolution clear native PAGE (hrCNE)

The protocol for hrCNE was adapted from published studies [71,72]. In summary, crude mt vesicles from ∼5x10^8^ cells were resuspended in a mt lysis buffer (2 mM ACA, 50 mM Imidazole-HCl, 1 mM EDTA, 50 mM NaCl, pH 7) and lysed for one hour on ice with 4 mg digitonin/1 mg protein. The samples were spun down at 16,000 g for 30 min and the cleared lysate protein concentrations were determined by a Bradford assay. Samples were mixed with a 5x loading dye (0.1% Ponceau-S, 50% glycerol) and loaded onto a 3%-12% native gradient gel. After electrophoresis (3 hr, 100 V, 4°C), the resolved mt lysates were transferred onto a nitrocellulose membrane (overnight, 20 V, 4°C) and probed with selected antibodies.

### Na_2_CO_3_ submitochondrial fractionation

Na_2_CO_3_ extraction of mt membranes was adapted from a previously published protocol [73]. Mt vesicles from 1x10^9^ cells were isolated by hypotonic lysis as described above. The resulting supernatant from the 25G needle homogenization step was kept as a cytosolic fraction (CYTO). The mt pellet was further treated with digitonin (80 μg/ml) for 15 min on ice to disrupt the mt outer membrane. The material was then cleared by centrifugation (12,000 g, 20 min, 4°C) and the pelleted mitoplasts were resuspended in 0.1 M Na_2_CO_3_ buffer (pH 11.5) and incubated for 30 min on ice. A final ultracentrifugation step (100,000 g, 4°C for 1 hr) performed in an SW50Ti rotor of a Beckman Instrument resulted in a supernatant comprised of proteins from the mt matrix (MX), including stripped peripheral membrane proteins, and a pellet containing integral proteins isolated from the mt membrane fraction (M).

### Glycerol gradient (GG) sedimentation

Hypotonically purified mt vesicles from ∼2.5x10^9^ cells were resuspended in a GG lysis buffer (10 mM Tris, pH 7.2, 10 mM MgCl_2_, 200 mM KCl, 1mM DTT) and lysed with 1% Triton X-100 (30 min, on ice). The lysates were cleared by a centrifugation step (2x 16,000 g, 30 min, 4°C) and the protein concentration was determined by a Bradford assay. Mt cleared lysates were resolved by ultracentrifugation (Beckman Instrument, SW40 rotor) at 38,000 g for 5 hr on 11ml 10–30% GG, which was poured manually or using the Gradient Station (Biocomp) according to the manufacturer’s protocol. The glycerol gradients were then fractionated either manually or with the Gradient station and 500μl fractions were stored at -70°C.

### Immunogold staining of ultrathin sections and transmission electron microscopy

Cells (∼5x10^7^) were pelleted (1300 g, 10 min, RT), washed in PBS (pH 7.4) and immediately fixed in a 4% formaldehyde/ 0.1 M phosphate buffer. Samples were then dehydrated at -10°C through a series of seven steps that increased the concentration of ethanol from 30% to100%, pausing at each step for 1 hour. Next, the samples were embedded in LR White Resin (Electron Microscopy Sciences) and polymerized (UV light, 48 hours at -10°C). Ultrathin sections were prepared by the Ultracut UCT ultramicrotome (Leica) and mounted on nickel grids. Prepared sections were blocked in 5% BSA, labelled with primary anti-β antibody (1:10 dilution), washed three times with PBS and incubated with protein A conjugated to 10 nm gold particles (1:100 dilution, Aurion). The immunogold labelled grids were contrasted, carbon coated and examined by the TEM (JEM-1010, Jeol). Grids, which were immunolabeled with only the protein A conjugated to 10nM gold particles, were used as a negative control.

### Quantification and statistical analysis of immunogold labelling

The number of gold particles was statistically evaluated as described earlier [12,74]. Using ImageJ software (NIH, USA), a grid consisting of squares (test points, P) with constant size (16,105 μm^2^) was superimposed randomly on the electron micrographs of identified mitochondria. All test squares and immunogold particles within the immediate proximity to the mt membrane were counted separately for each micrograph. In order to statistically evaluate the labelling, all observed gold particles (N_obs_) and all test points (P) from 113 images captured from NON, IND3 and IND5 samples were tallied. Expected numbers of gold particles (N_exp_) for each sample were calculated as (total sum N_obs_ x P)/total sum P. To determine if the difference between the observed and expected particles was significant and not due to random fluctuations, a Chi-squared analysis χ^2^ = (N_obs—_N_exp_)^2^/ N_exp_ was performed using GraphPad QuickCalcs calculator (www.graphpad.com/quickcalcs). In addition, the relative labeling index (RLI), where the predicted RLI = 1 for random labelling, was calculated as RLI = N_obs_/ N_exp_.

### Gene IDs for the genes and proteins mentioned in this study

ATPaseTb2 (Tb927.5.2930), ATPaseTb1 (Tb927.10.520), TbAAC (Tb927.10.14820/30/40), p18 (Tb927.5.1710), mtHsp70 (Tb927.6.3740), MRP1 (Tb927.11.1710), APRT (Tb927.7.1780), enolase (Tb427.10.2890), subunit β (Tb927.3.1380).

## Supporting Information

S1 Fig Bioinformatics analysis of ATPaseTb2. A) The multiple sequence alignment of ATPaseTb2 homologs from the order Kinetoplastida was performed by ClustalW on the following species (accession number, name): *Trypanosoma vivax* Y486 (TvY486_0502300, ATPaseTv2), *T*. *cruzi* Sylvio X10/1 (TCSYLVIO_010784, ATPaseTc2), *T*. *congolense* IL3000 (TcIL3000_5_3200, ATPaseTco2), *T*. *cruzi* CL Brener Non-Esmeraldo-like (TcCLB.506321.280, ATPaseTcB2), *T*. *cruzi* marinkellei strain B7 (Tc_MARK_9008, ATPaseTcm2), *T*. *brucei* TREU927 (Tb927.5.2930, ATPaseTb2), *L*. *tarentolae* Parrot-TarII (LtaP08.0840, ATPaseLt2), *L*. *mexicana* MHOM/GT/2001/U1103 (LmxM.08.1100, ATPaseLmx2), *L*.*major* strain Friedlin (LmjF.08.1100, ATPaseLm2), *L*. *infantum* JPCM5 (LinJ.08.1010, ATPaseLin2), *L*. *donovani* BPK282A1 (LdBPK_081010.1, ATPaseLd2), *L*. *braziliensis* MHOM/BR/75/M2904 (LbrM.08.0870, ATPaseLbr2), *Bodo saltans* (ATPaseBs2), *Strigomonas culicis* (STCU_02070, ATPaseSc2). Sequences were obtained from GeneDB database or from Welcome Trust Sanger centrum (*B*. *saltans* sequence). Numbers at the top indicate the amino acid positions in *T*. *vivax* ATPaseTv2. The mitochondrial targeting signal for ATPaseTb2 (MTS, green) was predicted by Mitoprot II v1.101. The homologous region (red) to Bs_sub d was determined using HHpred toolkit. B) The homology of ATPaseTb2 to subunit d (*B*. *taurus*) was based on HHpred, which utilizes the homology detection & structure prediction by HMM-HMM comparison. (http://toolkit.tuebingen.mpg.de) The alignments consist of one or more blocks with the following lines: ss_pred: query secondary structure as predicted by PSIPRED (upper case letters: high probability, lower case letters: low probability).Q query_name: query sequence Q Consensus: query alignment consensus sequence Quality of colum-column match: very bad =; bad—; neutral.; good +; very good |T Consensus: template alignment consensus sequence T templ_name: template sequence T ss_dssp: template secondary structure as determined by DSSP T ss_pred: template secondary structure as predicted by PSIPRED (upper case letters: high probability, lower case letters: low probability) The consensus sequence uses capital letters for amino acids that occur with > = 60% probability and lower case letters for amino acids that have > = 40% probability. For unconserved columns a tilde is used. The line in the middle shows the column score between the query and template amino acid distributions. It gives a valuable indication for the alignment quality. (A unit of column score corresponds approximately to 0.6 bits.)
**=**: very bad match column score below-1.5
**-**: bad match column score between-1.5 and-0.5
**.**: neutral match column score between -0.5 and +0.5
**+**: good match column score between +0.5 and +1.5
**|**: very good match column score above +1.5
(PDF)

S2 Fig Densitometric analysis of anti-sub_β and anti-p18 immunoblots of the BF ATPaseTb2 RNAi glycerol gradients depicted in Fig. 5D. The glycerol gradient fractions analyzed by western blot using anti-β (A) and anti-p18 (B) antibodies (Fig. 5D) were also examined using densitometry analysis. The chemiluminescent blots were imaged with the LAS3000 Imaging System (FUJI). The specific bands for subunit β and p18 were selected using the band analysis tool from the ImageQuant TL software (Amersham Biosciences), which allowed their background-subtracted densities to be determined. The background-corrected volumes of the corresponding protein bands were normalized to the highest value of each blot, which was set to 100.
(PDF)

S3 Fig ATPaseTb2 depletion alters the distribution of the F_1_-ATPase subunit β in *T*.*b*. ***evansi* mitochondria.** A) Ultrathin sections of RNAi non-induced cells (NON) and cells induced for 3 (IND3) and 5 (IND5) days were immunostained with a primary anti-β antibody, followed by incubation with a 10 nM gold bead conjugated anti-protein A secondary antibody. Images of the electron micrographs were captured and the immunogold particles visualized within identified mitochondria. Particles located in the matrix are marked with dashed arrows, while gold beads located within the immediate proximity of the mt membrane are designated with a solid arrow. B) All immunogold beads identified from 113 images of NON, IND3 and IND5 electron micrographs were itemized according to their localization and plotted as either mt inner membrane associated (grey) or matrix (white). C) Counts of observed mt membrane associated gold particles (N_obs_) and all test points (P) from NON, IND3 and IND5 images were recorded under their appropriate column. Expected numbers of gold particles (N_exp_) were calculated as (total sum N_obs_ x P)/total sum P. D) The relative labeling index was calculated (RLI = N_obs_/ N_exp_) for the mt membrane associated gold particles tabulated in S3B Fig. and is depicted on the y-axis of the column graph.
(PDF)
