# Supplementary figures and images for: ATPaseTb2, a Unique Membrane-bound FoF1-ATPase Component, Is Essential in Bloodstream and Dyskinetoplastic Trypanosomes
Source: PLoS Pathog. 2015 Feb 25;11(2):e1004660. doi: 10.1371/journal.ppat.1004660 (PMC4340940; doi:10.1371/journal.ppat.1004660)

A

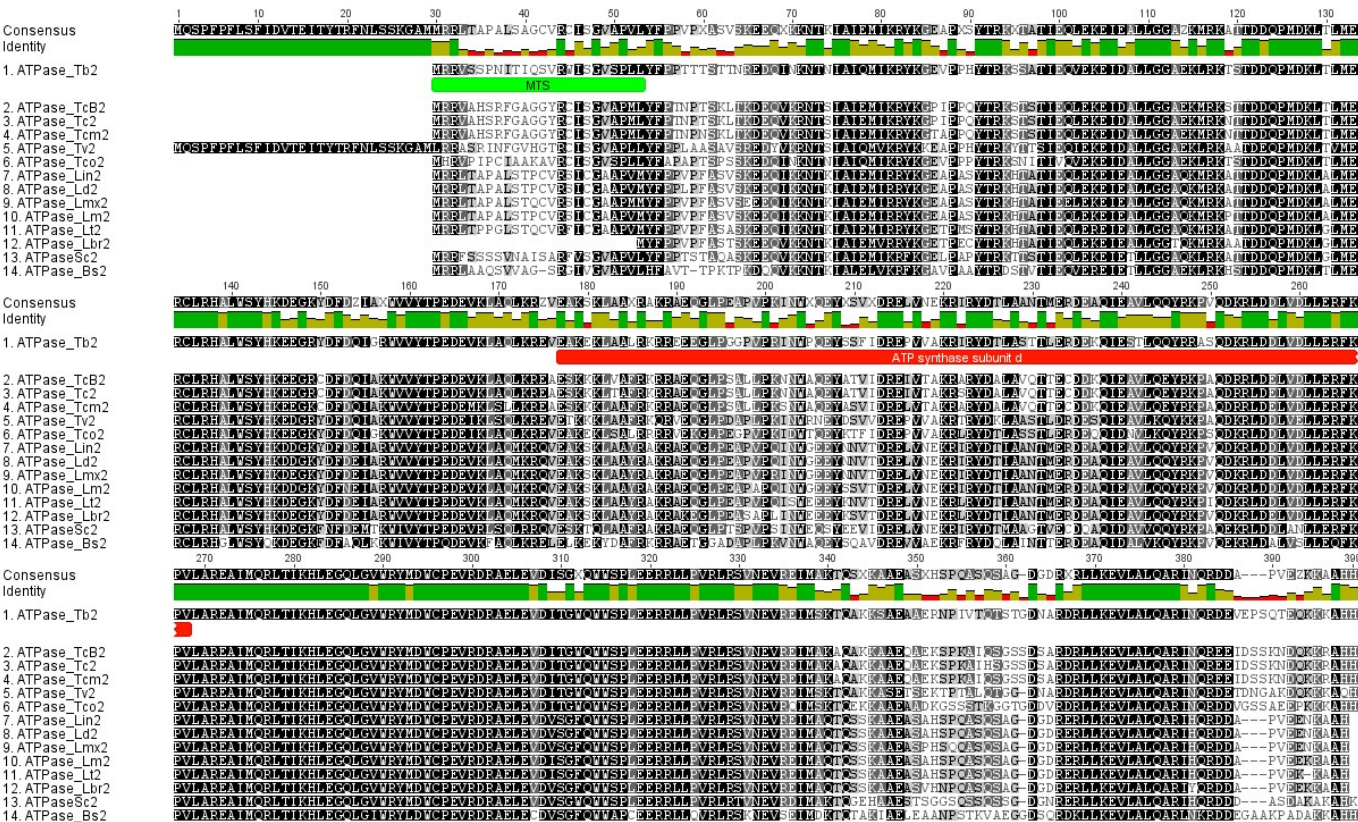

B

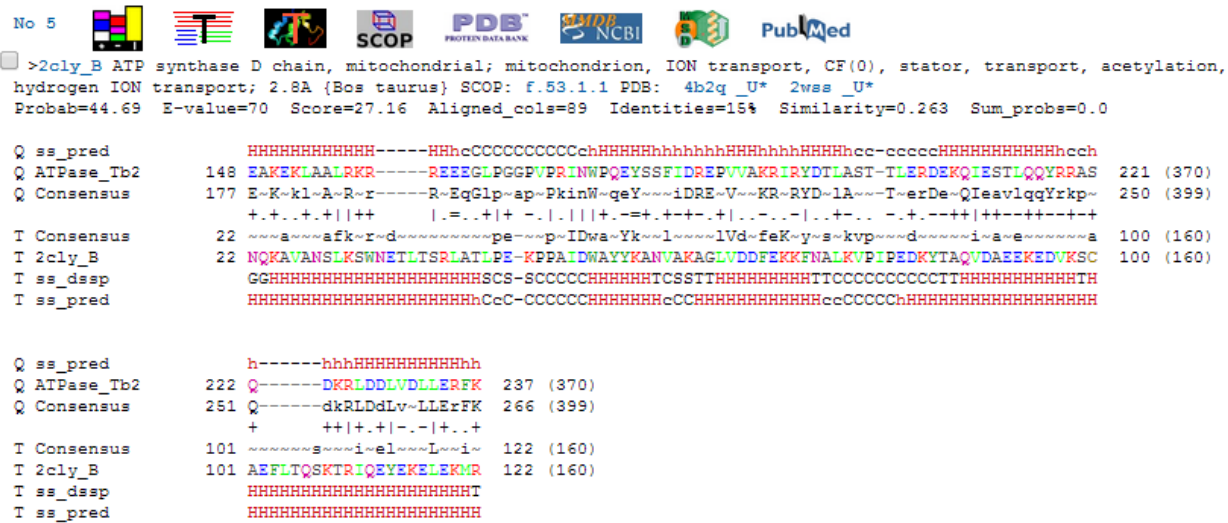

Supplement: S1 Fig — A) The multiple sequence alignment of ATPaseTb2 homologs from the order Kinetoplastida was performed by ClustalW on the following species (accession number, name): Trypanosoma vivax Y486 (TvY486_0502300, ATPaseTv2), T. cruzi Sylvio X10/1 (TCSYLVIO_010784, ATPaseTc2), T. congolense IL3000 (TcIL3000_5_3200, ATPaseTco2), T. cruzi CL Brener Non-Esmeraldo-like (TcCLB.506321.280, ATPaseTcB2), T. cruzi marinkellei strain B7 (Tc_MARK_9008, ATPaseTcm2), T. brucei TREU927 (Tb927.5.2930, ATPaseTb2), L. tarentolae Parrot-TarII (LtaP08.0840, ATPaseLt2), L. mexicana MHOM/GT/2001/U1103 (LmxM.08.1100, ATPaseLmx2), L.major strain Friedlin (LmjF.08.1100, ATPaseLm2), L. infantum JPCM5 (LinJ.08.1010, ATPaseLin2), L. donovani BPK282A1 (LdBPK_081010.1, ATPaseLd2), L. braziliensis MHOM/BR/75/M2904 (LbrM.08.0870, ATPaseLbr2), Bodo saltans (ATPaseBs2), Strigomonas culicis (STCU_02070, ATPaseSc2). Sequences were obtained from GeneDB database or from Welcome Trust Sanger centrum (B. saltans sequence). Numbers at the top indicate the amino acid positions in T. vivax ATPaseTv2. The mitochondrial targeting signal for ATPaseTb2 (MTS, green) was predicted by Mitoprot II v1.101. The homologous region (red) to Bs_sub d was determined using HHpred toolkit. B) The homology of ATPaseTb2 to subunit d (B. taurus) was based on HHpred, which utilizes the homology detection & structure prediction by HMM-HMM comparison. (http://toolkit.tuebingen.mpg.de) The alignments consist of one or more blocks with the following lines: ss_pred: query secondary structure as predicted by PSIPRED (upper case letters: high probability, lower case letters: low probability).Q query_name: query sequence Q Consensus: query alignment consensus sequence Quality of colum-column match: very bad =; bad—; neutral.; good +; very good |T Consensus: template alignment consensus sequence T templ_name: template sequence T ss_dssp: template secondary structure as determined by DSSP T ss_pred: template secondary structure as predicted by PSI [file ppat.1004660.s001.pdf]

A

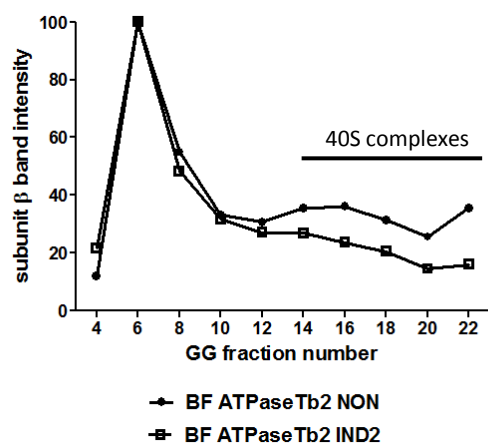

B

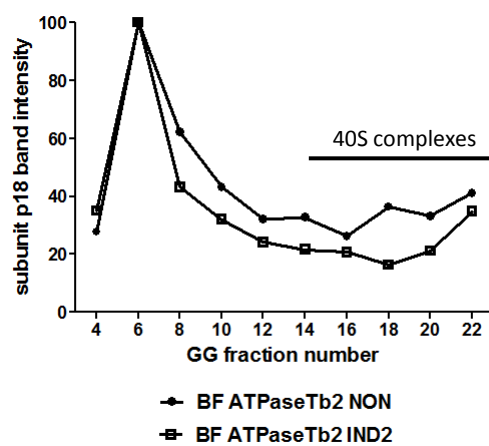

Supplement: S2 Fig — The glycerol gradient fractions analyzed by western blot using anti-β (A) and anti-p18 (B) antibodies (Fig. 5D) were also examined using densitometry analysis. The chemiluminescent blots were imaged with the LAS3000 Imaging System (FUJI). The specific bands for subunit β and p18 were selected using the band analysis tool from the ImageQuant TL software (Amersham Biosciences), which allowed their background-subtracted densities to be determined. The background-corrected volumes of the corresponding protein bands were normalized to the highest value of each blot, which was set to 100. (PDF) [file ppat.1004660.s002.pdf]
