# Supplementary material for: ATPaseTb2, a Unique Membrane-bound FoF1-ATPase Component, Is Essential in Bloodstream and Dyskinetoplastic Trypanosomes
Source: PLoS Pathog. 2015 Feb 25;11(2):e1004660. doi: 10.1371/journal.ppat.1004660 (PMC4340940; doi:10.1371/journal.ppat.1004660)
Supplement: S3 Fig — evansi mitochondria. A) Ultrathin sections of RNAi non-induced cells (NON) and cells induced for 3 (IND3) and 5 (IND5) days were immunostained with a primary anti-β antibody, followed by incubation with a 10 nM gold bead conjugated anti-protein A secondary antibody. Images of the electron micrographs were captured and the immunogold particles visualized within identified mitochondria. Particles located in the matrix are marked with dashed arrows, while gold beads located within the immediate proximity of the mt membrane are designated with a solid arrow. B) All immunogold beads identified from 113 images of NON, IND3 and IND5 electron micrographs were itemized according to their localization and plotted as either mt inner membrane associated (grey) or matrix (white). C) Counts of observed mt membrane associated gold particles (Nobs) and all test points (P) from NON, IND3 and IND5 images were recorded under their appropriate column. Expected numbers of gold particles (Nexp) were calculated as (total sum Nobs x P)/total sum P. D) The relative labeling index was calculated (RLI = Nobs/ Nexp) for the mt membrane associated gold particles tabulated in S3B Fig. and is depicted on the y-axis of the column graph. (PDF) [file ppat.1004660.s003.pdf]

Figure S3

A

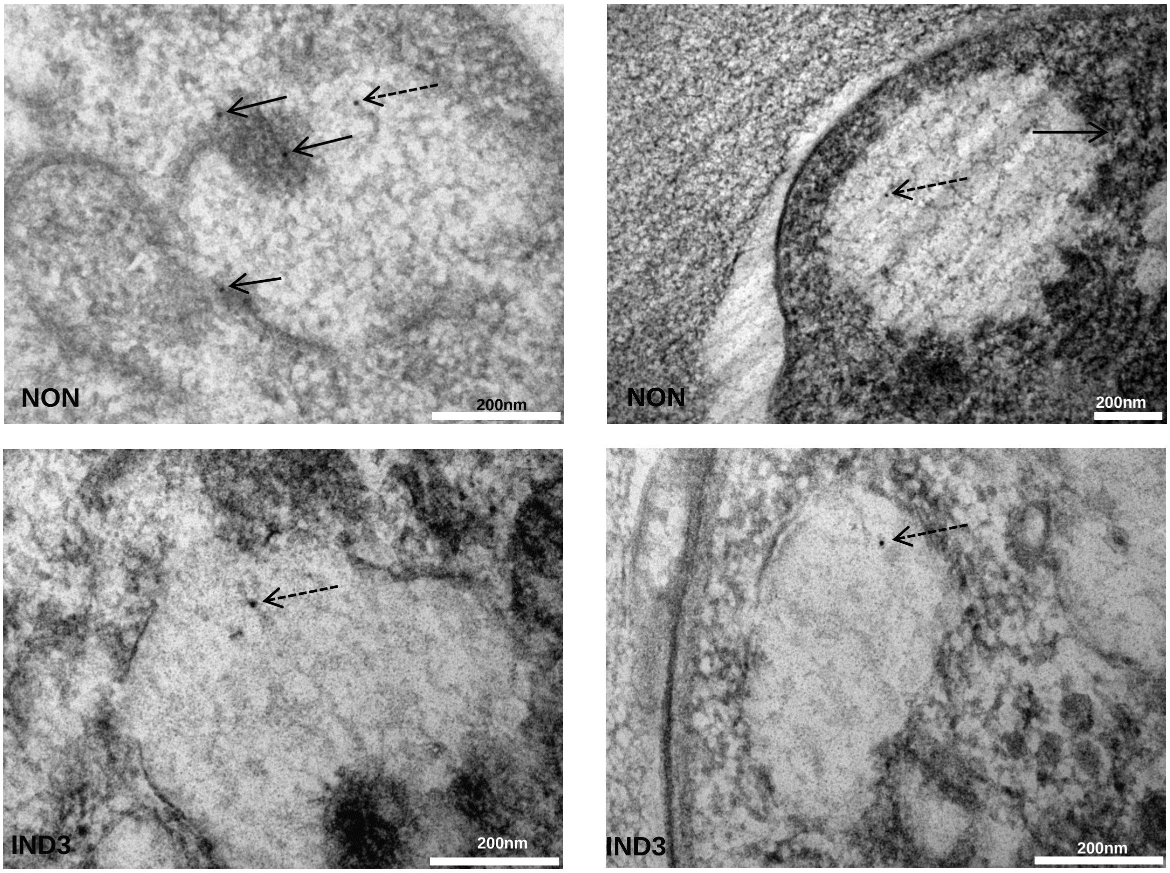

B

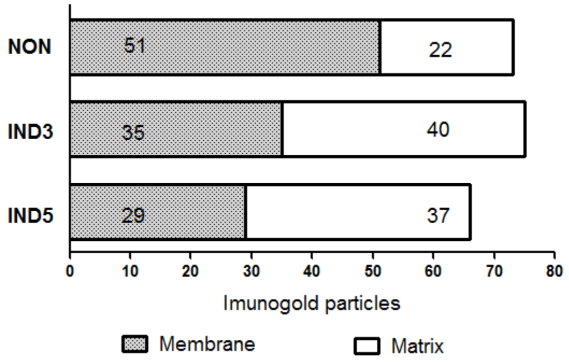

D

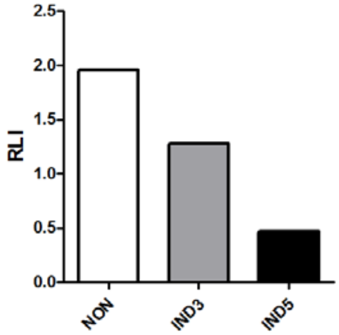

C

	Gold particles observed (N_{obs})	Test point number (P)	Gold particles expected (N_{exp})	χ2 partial value
**NON**	51.0	1226.0	26.0	24.0
**IND3**	35.0	1287.0	27.3	2.2
**IND5**	29.0	2906.0	61.7	17.3
**total**	**115.0**	**5419.0**	**115.0**	**43.5**
